# Structural and functional studies of legumain–mycocypin complexes revealed a competitive, exosite-regulated mode of interaction

**DOI:** 10.1016/j.jbc.2022.102502

**Published:** 2022-09-16

**Authors:** Tasneem Elamin, Naiá P. Santos, Peter Briza, Hans Brandstetter, Elfriede Dall

**Affiliations:** Department of Biosciences and Medical Biology, University of Salzburg, Salzburg, Austria

**Keywords:** cysteine protease, protease inhibitor, crystal structure, structural biology, pH regulation, protein stability, asparaginyl endopeptidase, ligase, clitocypin, macrocypin, AAN-AMC, Z-Ala-Ala-Asn-7-amino-4-methylcoumarin, ACP, asparaginyl carboxypeptidase, AEP, asparaginyl endopeptidase, Clt2, clitocypin 2, Kgp, gingipain K, Mcp1a, macrocypin 1a, Mcp2a, macrocypin 2a, Mcp3a, macrocypin 3a, Mcp4a, macrocypin 4a, MMTS, S-methyl methanethiosulfonate, RCL, reactive center loop, SEC, size-exclusion chromatography

## Abstract

Under pathophysiologic conditions such as Alzheimer’s disease and cancer, the endolysosomal cysteine protease legumain was found to translocate to the cytosol, the nucleus, and the extracellular space. These noncanonical localizations demand for a tight regulation of legumain activity, which is in part conferred by protein inhibitors. While there is a significant body of knowledge on the interaction of human legumain with endogenous cystatins, only little is known on its regulation by fungal mycocypins. Mycocypins are characterized by (i) versatile, plastic surface loops allowing them to inhibit different classes of enzymes and (ii) a high resistance toward extremes of pH and temperature. These properties make mycocypins attractive starting points for biotechnological and medical applications. In this study, we show that mycocypins utilize an adaptable reactive center loop to target the active site of legumain in a substrate-like manner. The interaction was further stabilized by variable, isoform-specific exosites, converting the substrate recognition into inhibition. Additionally, we found that selected mycocypins were capable of covalent complex formation with legumain by forming a disulfide bond to the active site cysteine. Furthermore, our inhibition studies with other clan CD proteases suggested that mycocypins may serve as broad-spectrum inhibitors of clan CD proteases. Our studies uncovered the potential of mycocypins as a new scaffold for drug development, providing the basis for the design of specific legumain inhibitors.

The human cysteine protease legumain is mainly localized to the endolysosomal system where it represents a key enzyme for the processing of (self-)antigens for presentation on the MHCII complex (1). Its marked preference for cleaving after asparagine residues led to its synonymous naming as the asparaginyl endopeptidase (AEP) (2, 3, 4, 5, 6). By sequence comparison, legumain belongs to clan CD and family C13 of cysteine proteases, indicating its relation to the caspases (7, 8). Legumain is synthesized as an inactive zymogen, harboring an N-terminal caspase-like catalytic domain and a C-terminal death-domain–like prodomain (9). Activation of prolegumain proceeds *via* the electrostatic release of the prodomain during endolysosomal maturation. While the proenzyme is stable at neutral pH, the isolated AEP domain is stable only at acidic pH (9, 10). In addition to its well-established protease activity, legumain also harbors a pH-dependent ligase activity, that is, legumain can also synthesize peptide bonds (11, 12). Besides its important immunological function, legumain is also a key player in the activation of endolysosomal cathepsins and of Toll-like receptors (13, 14, 15, 16). Moreover, in a number of human solid tumors and in the aged brain, legumain was found overexpressed and translocated to the extracellular space, the nucleus, or the cytosol, thereby facilitating tumor growth and neuronal damage (17, 18). Its relevance in tumor progression and neurodegenerative disorders reflects its importance as a diagnostic cancer marker and potential therapeutic target (2, 19, 20, 21, 22). Since dysregulation of legumain activity can lead to severe disorders, specific regulators of its enzymatic activities are much sought after. Regulating factors known so far include pH, substrate specificity, binding to integrin α_V_β_3_, its prodomain, peptidic inhibitors, small molecule inhibitors, and naturally occurring protein inhibitors of the cystatin and mycocypin families (9, 23, 24). Mycocypins are a family of fungal (cysteine) protease inhibitors with so far two known members: clitocypin (isolated from the cloud funnel *Clitocybe nebularis*) and macrocypin (isolated from the parasol *Macrolepiota procera*) (25, 26). Under physiologic conditions, they play important functions in the defense against exogenous proteases during pathogen infection. Crystal structures of clitocypin and macrocypin 1 have been solved and revealed a β-trefoil fold, reminiscent of a tree (27). The tree is built up by a short trunk formed by a six-stranded β-barrel and a crown providing the versatile surface, which allows them to inhibit several classes of proteases: papain-like enzymes, legumain, and trypsin. Inhibition constants of macrocypins and clitocypin toward legumain are in the nM range (K_I_: Mcp1: 3.3 nM; Clt: 21.5 nM) (27). In agreement with mutagenesis data, the structures suggest disjunct papain and legumain reactive sites, functionally resembling the situation of cystatins. The proposed legumain reactive center loop (RCL) harbors a conserved asparagine residue, which likely serves as P1 residue, similar as in cystatins (27, 28). The mycocypin fold is further characterized by high stability toward extremes of pH, denaturants, and temperature and is capable of reversible unfolding (29, 30). Because of their unique properties, mycocypins provide high potential which might be exploited in crop protection (*e.g.*, in potatoes) (31, 32), for medical applications (engineered inhibitors) and on a more fundamental level, to study enzymatic mechanisms. How mycocypins are interacting with legumain was not studied in detail so far. Therefore, we set out to structurally and biochemically analyze their mode of interaction.

## Results

### Crystal structures of legumain in complex with mycocypins

**Figure 1:**
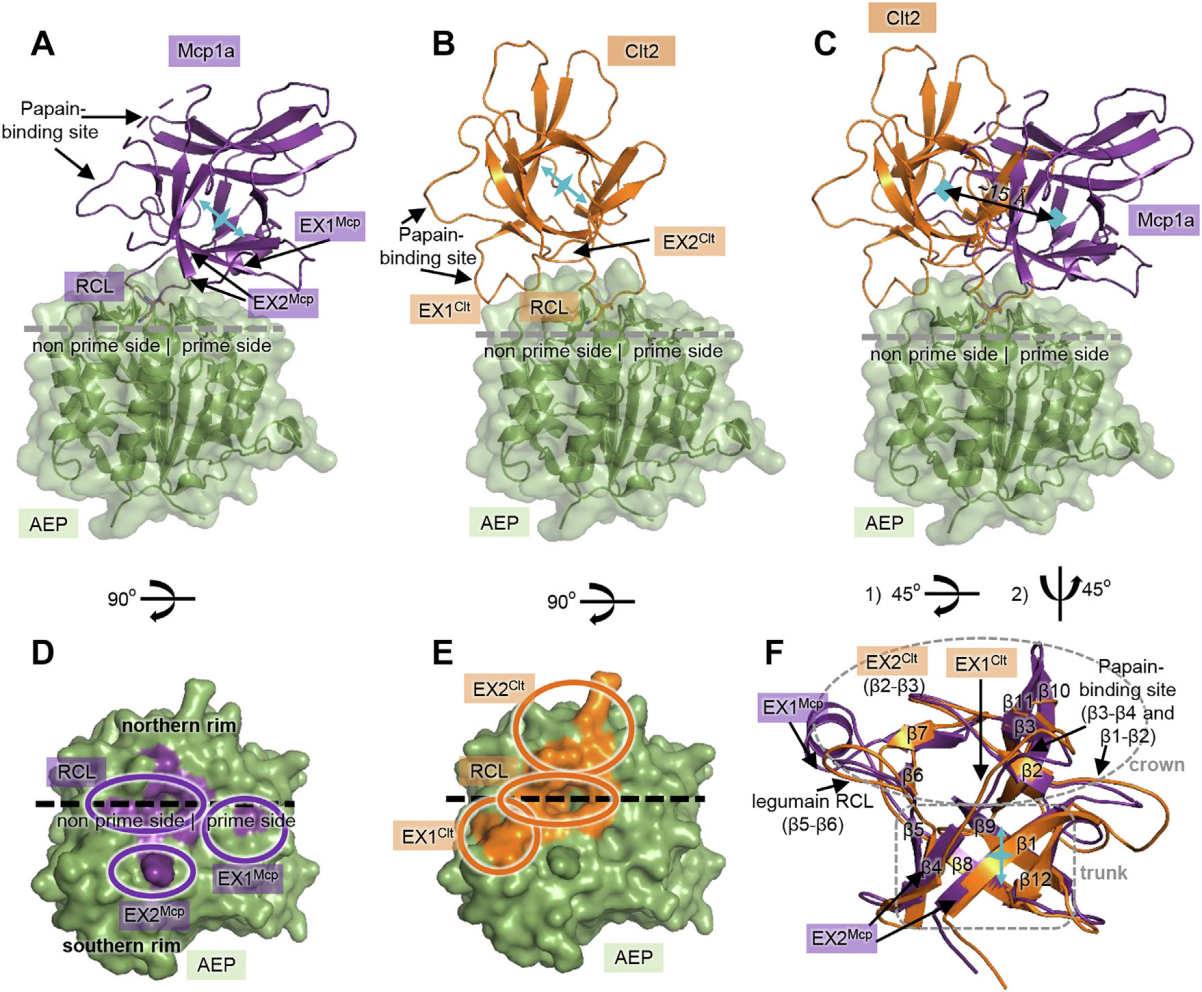
**Crystal structures of legumain in complex with macrocypin 1a and clitocypin 2 revealed active site and exosite interactions.***A*, cocrystal structure of human legumain (*green surface*; AEP: asparaginyl endopeptidase) in complex with Mcp1a (*purple cartoon*). The P1-Asn74^Mcp^ residue on the Mcp1a reactive center loop (RCL) is shown as *purple sticks* and indicates the localization of the active site. *B*, cocrystal structure of human legumain in complex with Clt2 (*orange cartoon*). The P1-Asn70^Clt^ residue on the RCL is shown in *orange sticks*. *C*, superposition of the legumain–Mcp1a and legumain–Clt2 complex structures. *D* and *E*, view on the legumain active site in standard orientation, rotated by 90° relative to *A* and *B*. The substrate-binding site is indicated by a *dashed line*. A substrate would bind from *left* to *right*. Regions interacting with the inhibitor are colored in *purple* (Mcp1a) or *orange* (Clt2), respectively. *F*, superposition of Mcp1a (*purple*) and Clt2 (*orange*). Both mycocypins harbor a β-trefoil fold, formed by the 6-stranded β-barrel. Mycocypins have a tree-like architecture where the trunk is formed by the short β-barrel and the crown by the connecting loops. The orientation of the trunk axis in (*F*) relative to (*A*), (*B*), and (*E*) is indicated in *light blue*. Interaction sites are indicated. Clt2, clitocypin 2; Mcp1a, macrocypin 1a.

To get a detailed understanding of how mycocypins interact with legumain, we solved the crystal structure of human legumain in complex with *M. procera* macrocypin 1a (Mcp1a) and *C. nebularis* clitocypin 2 (Clt2), representatives of the macrocypin and clitocypin families, respectively. We selected the human legumain variant as a target enzyme because it is especially interesting for drug development. Mcp1a and Clt2 were recombinantly expressed in *Escherichia coli* and purified from nonclassical inclusion bodies or as soluble protein, respectively. To prevent undesired proteolytic processing, we covalently blocked the active site cysteine Cys189 of legumain with S-methyl methanethiosulfonate (MMTS), before cocrystallizing legumain in complex with Mcp1a or Clt2. We solved the crystal structure of legumain in complex with Mcp1a to a resolution of 2.2 Å in space group C222_1_, with two legumain–Mcp1a complexes in the asymmetric unit (Table 1, Figs. 1*A* and S1*A*). Overall, the structures of both legumain and Mcp1a within the complex looked similar as compared to their free forms. No major conformational changes had occurred upon complex formation. The structure furthermore showed that the interaction of Mcp1a with legumain was mediated *via* three major regions on the inhibitor (Fig. 1*D*). We identified (i) the RCL (loop β5-β6) that is interacting with the active site of legumain, (ii) exosite 1 (EX1^Mcp^) that is mediating ionic interactions to the prime substrate interaction sites on legumain, and (iii) exosite 2 (EX2^Mcp^) that is mediating hydrophobic interactions to the area south of the nonprime substrate-binding site. Furthermore, we resolved the crystal structure of legumain in complex with Clt2 to a resolution of 1.8 Å in space group P2_1_ and with one enzyme-inhibitor assembly in the asymmetric unit (Figs. 1*B* and S1*B*). Similarly, the overall fold of legumain and Clt2 remained largely unchanged in the complex. No major conformational changes were observed within the complex. Clt2 was binding to legumain *via* three primary interaction sites (Fig. 1*E*): (i) A reactive center loop which is establishing the interaction to the legumain active site, (ii) exosite 1 (EX1^Clt^) that is mediating further interactions to the nonprime substrate-interaction sites, and (iii) exosite 2 (EX2^Clt^) which is mediating interactions to the area north of the nonprime substrate-binding cleft. Overall, both inhibitors show the same β-trefoil fold that is reminding of a tree (Fig. 1*F*). In this picture, the tree is built up by a short trunk formed by a six-stranded β-barrel and a crown harboring the interaction site for papain-like enzymes (loops β1-β2 and β3-β4) and the legumain RCL (β5-β6). Interestingly, even though Mcp1a and Clt2 shared a high structural similarity (topmatch calculated rmsd using Cα atoms: 1.6 Å) and exploited a similar mode of interaction with legumain dominated by an RCL, they differed dramatically in their exosite interactions. While the Mcp1a exosites predominantly interacted with the area south of the substrate-binding cleft and the prime side, the Clt2 exosite interaction was localized to the north of the substrate-binding cleft and the nonprime-interaction sites on legumain. Consistent with these largely disjunct Mcp1a and Clt2 exosites, they also mapped to different areas on the inhibitors. While exosites 1 and 2 on Mcp1a localized to its trunk and crown, both Clt2 exosites localized to the crown. Consequently, the trunk regions of Mcp1a and Clt2 were shifted by approximately 15 Å relative to each other and did not superpose onto each other when complexed with legumain (Fig. 1*C*).

**Table 1:** X-ray data collection and refinement statistics

	AEP-Mcp1a (pdb 8AE5)	AEP-Clt2 (pdb 8AE4)
Data collection		
Space group	*C222(1)*	*P12(1)1*
Cell dimensions		
*a*, *b*, *c* (Å)	159.1, 174.1, 112.9	42.4, 63.6, 85.5
*α*, *β*, *γ* (°)	90, 90, 90	90, 102, 90
Resolution (Å)^a^	40.72–2.29 (2.37–2.29)	41.82–1.79 (1.90–1.79)
*R*_meas_	0.11 (3.83)	0.14 (1.46)
*CC (1/2) (%)*	0.99 (0.33)	0.99 (0.49)
*I*/σ*I*	13.7 (0.5)	11.2 (1.1)
Completeness (%)	99.1 (94.2)	97.1 (80.5)
Refinement		
Resolution (Å)	40.7–2.29	41.8–1.79
No. of reflections	70,029	40,876
*R*_work_/*R*_free_	22.1/25.8	19.2/20.7
No. of atoms		
Protein	6849	3316
Ligand/ion	125	56
Water	80	325
Overall B-factor (Å^2^)	93.6	26.9
R.m.s deviations		
Bond lengths (Å)	0.009	0.007
Bond angles (º)	1.08	0.93

The structure was determined from a single crystal.

a Highest resolution shell is shown in parentheses.

### The RCL binds in a substrate-like manner to the active site

**Figure 2:**
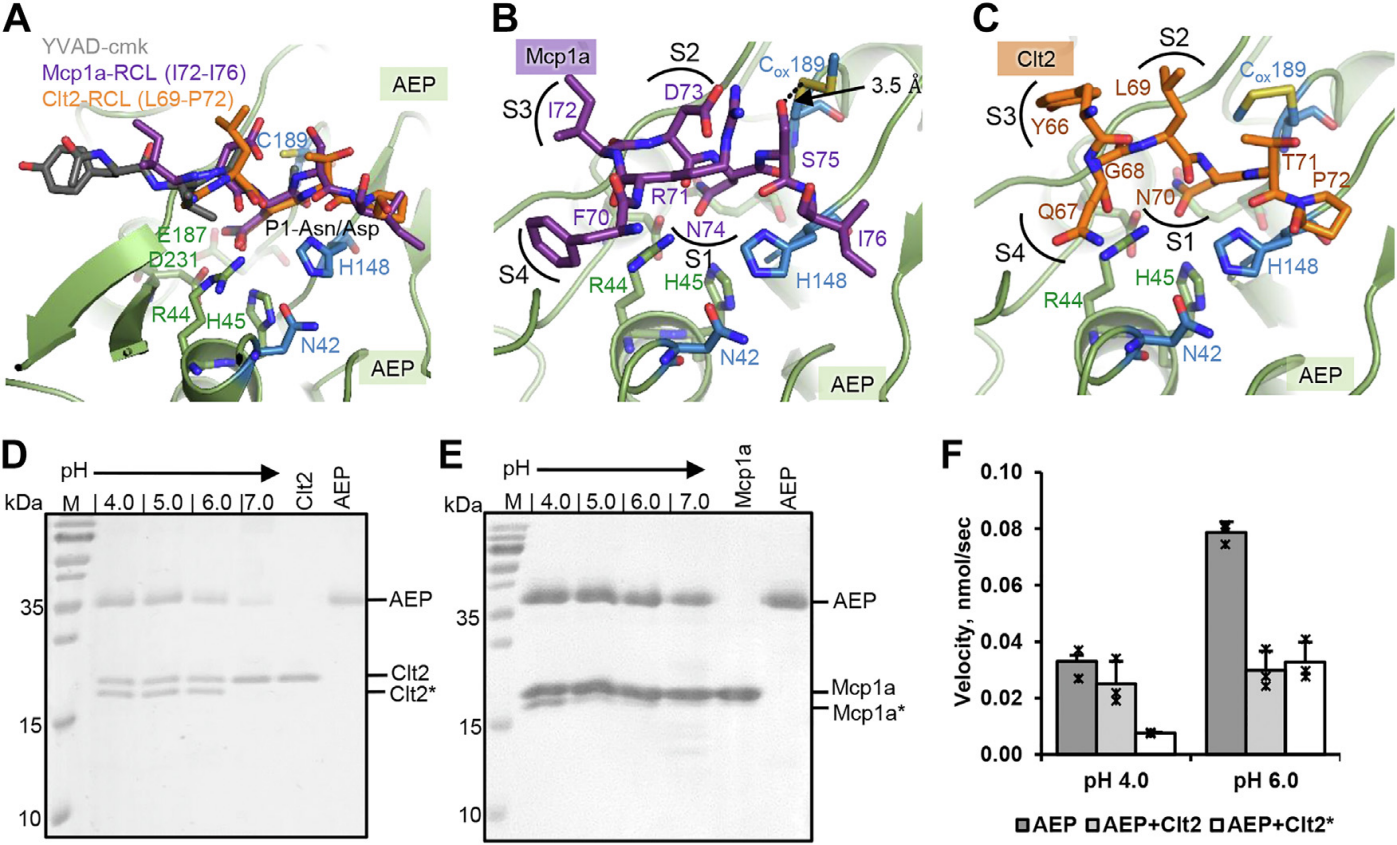
**Mycocypins show a substrate-like mode of inhibition.***A*, zoom-in view on the legumain active site, after superposition of the legumain–Mcp1a and legumain–Clt2 complex structures with YVAD-cmk–inhibited legumain (pdb 4aw9). Legumain is shown in *green cartoon* (AEP), the YVAD-cmk inhibitor in *gray sticks*, and the Mcp1a and Clt2 RCLs in *purple* and *orange sticks*, respectively. Catalytic residues on legumain are shown as *blue sticks* and residues forming the S1-pocket in *green sticks*. *B* and *C*, zoom-in view on the legumain active site (*green*) bound to Mcp1a (*purple*, *B*) or Clt2 (*orange*, *C*). Catalytic residues are shown as *blue sticks*, residues forming the S1-pocket as *green sticks*, and residues belonging to the Clt2 or Mcp1a RCL as *orange* or *purple sticks*, respectively. The catalytic Cys189 residue is modified with MMTS (C_ox_189). *D*, coincubation of legumain with Clt2 in a 1:2 molar ratio at pH 4.0 to 7.0 revealed cleavage at pH 4.0 to 6.0 (Clt2∗). *E*, coincubation of legumain with Mcp1a in a 1:2 molar ratio at pH 4.0 to 7.0 revealed cleavage only at pH 4.0 (Mcp1a∗). *F*, enzymatic activity of 2 nM legumain (AEP) after incubation with 10 nM of intact Clt2 and processed Clt2∗, measured as turnover of the AAN-AMC substrate at pH 4.0 and 6.0. AAN-AMC, Z-Ala-Ala-Asn-7-amino-4-methylcoumarin; AEP, asparaginyl endopeptidase; Clt2, clitocypin 2; Mcp1a, macrocypin 1a; MMTS, S-methyl methanethiosulfonate; RCL, reactive center loop.

Superposition of the complex structures with the Ac-Tyr-Val-Ala-Asp-chloromethylketone (YVAD-cmk)-bound legumain structure (pdb 4aw9) confirmed a substrate-like binding mode of the RCL’s of both inhibitors (Fig. 2*A*). We identified Asn74^Mcp^ (^Mcp^ denotes Mcp1a numbering) and Asn70^Clt^ (^Clt^ denotes Clt2 numbering) as P1 residues on the Mcp1a and Clt2 RCLs, respectively (Figs. 2*A* and S2). Sequence analysis furthermore confirmed that the P1-Asn residues are strictly conserved within the legumain inhibitory mycocypins (Fig. S3). Additionally, we found that Phe70^Mcp^, Ile72^Mcp^, and Asp73^Mcp^ on the Mcp1a RCL serve as P4, P3, and P2 residues, respectively (Fig. 2*B*). Similarly, the RCL of Clt2 provides interactions to the S4–S1 substrate-binding sites. Specifically, Gln67^Clt^ served as P4 residue, the side chain of Tyr66^Clt^ and the main chain of Gly68^Clt^ as P3 residue, and Leu69^Clt^ as P2 residue in Clt2 (Fig. 2*C*). Our structural analysis revealed that both Mcp1a and Clt2 bound to legumain in a substrate-like manner, using a conserved P1-Asn on the RCL. This observation further suggested that the inhibitors would get hydrolyzed upon binding to legumain. To test whether mycocypins are substrates to the legumain protease activity, we coincubated them with legumain at pH 4.0 to 7.0 in a 1:2 molar ratio (enzyme:inhibitor) and analyzed the reaction for cleavage products using SDS-PAGE and mass spectrometry assays. Indeed, we found that Clt2 was efficiently cleaved by legumain at pH 4.0 to 6.0 (Fig. 2*D*). When the samples analyzed by SDS-PAGE were not heated before loading on the gel, the cleavage product was visible as a band migrating slightly faster than the intact inhibitor. If the samples were heated before loading them on the gel, the band corresponding to the intact inhibitor was shifted downward and the cleavage products migrated as two distinct bands, corresponding to the N-terminal Met1^Clt^–Asn70^Clt^ and C-terminal Thr71^Clt^–His160^Clt2^ (including the C-terminal His_6_-tag) fragments (Fig. S4, *A* and *C*). These differences in migration behavior suggested that without heating, Clt2 would remain (partly) folded on SDS-PAGE. Mass spectrometry analysis confirmed Asn70^Clt^ on the RCL as the predominant processing site (Fig. S4*E*), providing further evidence that Asn70^Clt^ acts as a P1-residue. Interestingly, when we repeated the experiment using Mcp1a, we observed cleavage of the inhibitor only at pH ≤ 4.0 (Fig. 2*E*). Similarly, when we analyzed the samples by SDS-PAGE without heating them, the cleavage product was visible as a single band migrating below the intact inhibitor. Heating of the samples led to complete denaturation, associated with a shift of intact Mcp1a to an apparent higher molecular weight and migration of the cleavage products as two distinct bands. These bands corresponded to the N-terminal Met1^Mcp^–Asn74^Mcp^ and C-terminal Ser75^Mcp^–His177^Mcp^ (including the C-terminal His6-tag) fragments (Fig. S4, *B* and *C*). Mass spectrometry experiments confirmed that the predominant cleavage site was the P1-residue Asn74^Mcp^ on the RCL (Fig. S4*F*). In a next step, we were wondering whether the inhibitors remained bound to legumain after they were processed. To answer this question, we subjected the cleavage reactions to size-exclusion chromatography (SEC) experiments. Indeed, we found comigration of the cleavage products with legumain on SEC and subsequent SDS-PAGE analysis (Fig. S5, *A* and *B*). Given these results, we hypothesized that inhibitor processing was critical for the inhibition mechanism exploited by mycocypins. To further investigate the relevance of processing for inhibition, we tested the inhibitory activity of intact Clt2 and processed Clt2 (Clt2∗) toward legumain. To that end, we incubated legumain with a 4-fold excess of Clt2 at pH 5.5 for 4 h. Subsequently, we separated the processed, excess Clt2∗ from the legumain-Clt2 complex by SEC. When we tested inhibition of legumain by processed Clt2∗ and intact Clt2 at pH 4.0 and pH 6.0, we found that while both variants showed similar inhibition at pH 6, the cleaved inhibitor was more potent at acidic pH (Fig. 2*F*). This observation further supported our hypothesis that processing is indeed important for the inhibitory mechanism. It is turning the inhibitor even more potent at acidic pH.

Along the same line, we were wondering whether the processed inhibitor was also a substrate to the legumain ligase activity. In a previous study, we observed that cystatin E, an endogenous inhibitor of human legumain, was processed and religated by legumain in a pH-dependent manner (33). To test whether this was also the case for the mycocypins, we incubated legumain with Mcp1a or Clt2 at pH 4.0 to achieve quantitative (>50%) cleavage of the inhibitor. Subsequently, we shifted pH to 7.0 or added MMTS to trigger the ligase reaction (Fig. S5*C*). Interestingly, we did not observe conversion of the processed inhibitors to the intact precursors at the investigated conditions, which suggested that the processed mycocypins are unfavorable substrates to the legumain ligase activity.

### Active site interactions are conformationally controlled

**Figure 3:**
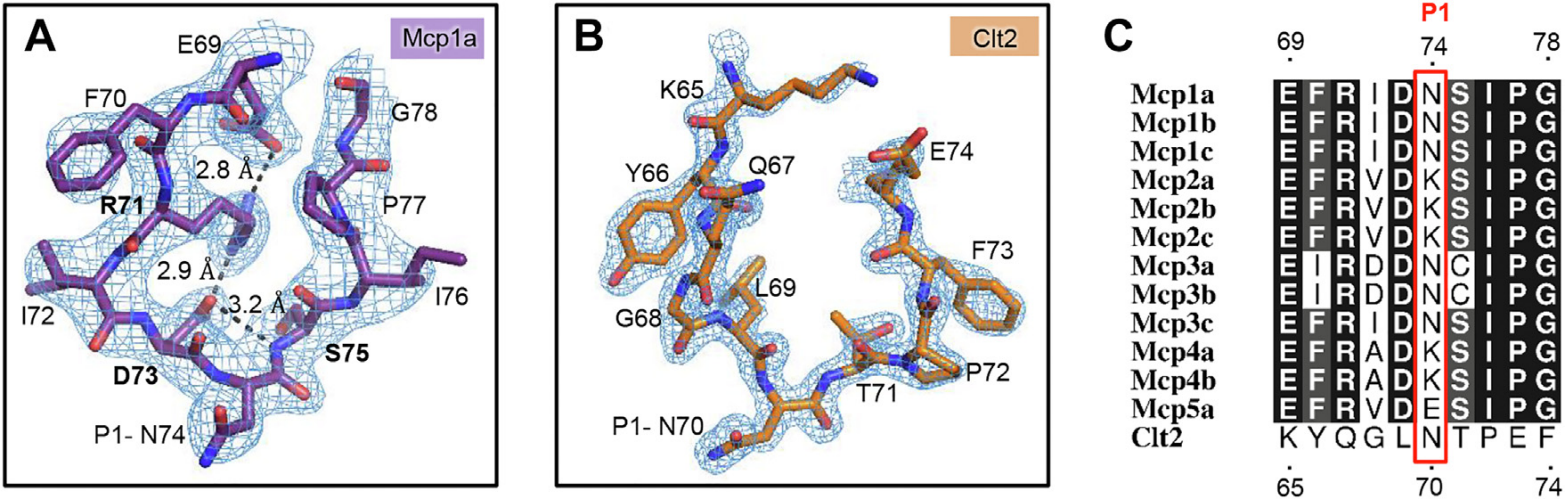
**The RCL conformation is stabilized in Mcp1a but flexible in Clt2.** The RCL’s of Mcp1a (*A*) and Clt2 (*B*) are shown as *purple* or *orange sticks*, respectively. Ionic and hydrogen bonding interactions stabilizing the RCL of Mcp1a are indicated as *dashed lines*. A 2Fo-Fc composite omit electron density map contoured at 2 σ over the mean is shown surrounding the RCL sequences. *C*, sequence alignment of Mcp and Clt2 RCL sequences. Details concerning sequences and programs used are specified in the M&M section. Clt2, clitocypin 2; Mcp1a, macrocypin 1a; RCL, reactive center loop.

While Clt2 was processed by legumain at acidic to near-neutral pH conditions, Mcp1a was only hydrolyzed at pH ≤ 4.0. To understand how these differences in interaction arise, we had a closer look on the RCL structures of Mcp1a and Clt2. Interestingly, we found that Mcp1a is internally stabilized by ionic interactions of P2-Asp73^Mcp^ and P6-Glu69^Mcp^ to P4-Arg71^Mcp^ and Ser75^Mcp^ to Asp73^Mcp^ (Fig. 3*A*). These interactions are conserved throughout the known macrocypin sequences (Figs. S3 and 3*C*). Contrasting the situation in Mcp1a, the RCL structure of Clt2 was missing an internal, stabilizing electrostatic clamp (Fig. 3*B*). This lacking clamp likely renders the Clt2 RCL conformation more flexible in solution, unlike the Mcp1a RCL. This conclusion is further supported by the absence of electron density of the RCL in the crystal structure of isolated Clt2 (pdb 3h6r). The RCL of Clt2 is, however, well defined when bound to legumain (Fig. 3*B*). At acidic pH, where we observed processing of the Mcp1a RCL, the internal electrostatic clamp will be disturbed due to protonation of Asp73^Mcp^ and Glu69^Mcp^, which could provide an explanation for the pH-dependent differences in processing we observed for Mcp1a and Clt2. Protonation at pH 4.0 may lead to an increase in Mcp1a RCL flexibility, similar as we find it in the RCL of Clt2. Together, this led us to the hypothesis that the increase in RCL flexibility at acidic pH would turn Mcp1a into a better protease substrate. To test the relevance of the RCL conformation for inhibition, we prepared Mcp1a-D73A and Mcp1a-R71E RCL-mutants. In agreement with our hypothesis, these mutants showed a decrease in inhibition and an increase in RCL hydrolysis (Table 2 and Fig. S6), confirming that not only the RCL sequence but also its conformation are critical for inhibition.

**Table 2:** Inhibition constants of Mcp1a and Clt2 variants toward human legumain

	K_i_ (nM)
Macrocypin 1a	
Mcp1a WT	2.18 ± 0.35
Mcp1a-D73A	113 ± 7
Mcp1a-R71E	147 ± 20
Mcp1a-R96A	183 ± 14
Clitocypin 2	
Clt2 WT	79 ± 3
Clt2-3x	6686 ± 384

### Cysteine in P1′ position mediates covalent complex formation

**Figure 4:**
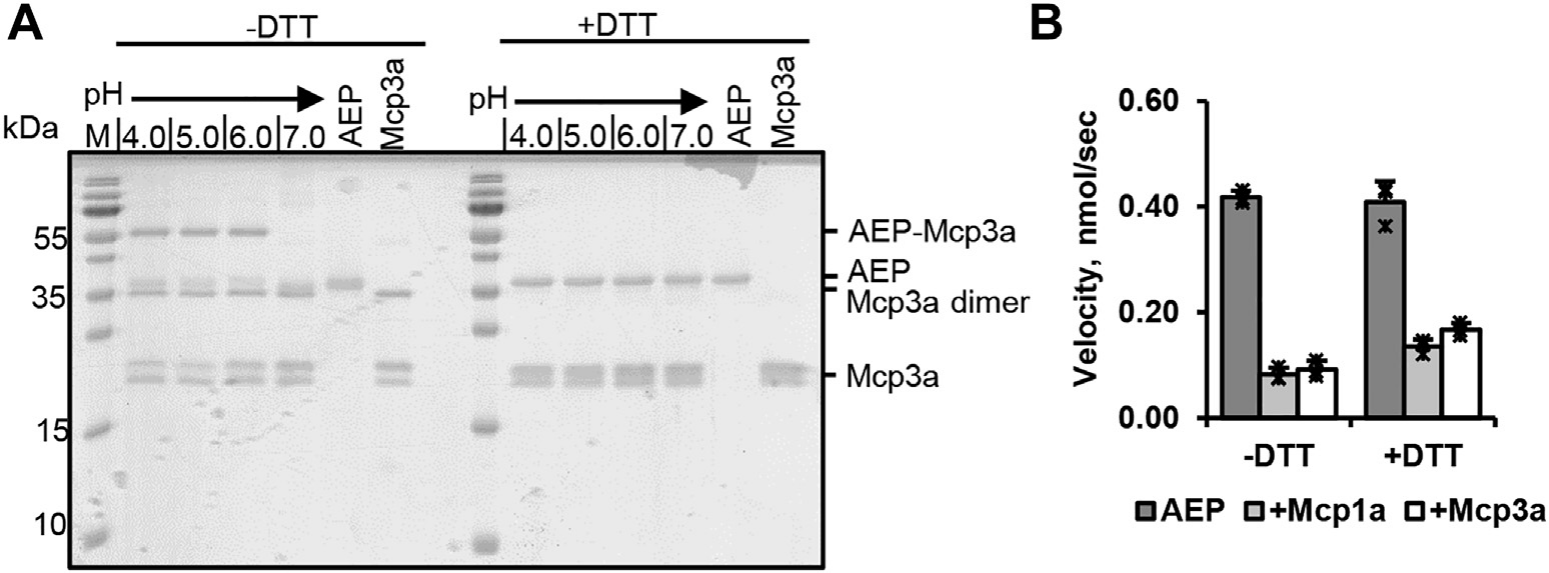
**Mcp3a is a covalent legumain inhibitor.***A*, coincubation of legumain (AEP) with Mcp3a at pH 4.0 to 7.0 led to the formation of a band migrating at the expected height of a covalent complex in the absence of reducing agent. The band was resolved into free AEP and Mcp3a when DTT was added to the sample loading buffer. In addition to monomeric Mcp3a, we also observed a band corresponding to dimeric Mcp3a, which was also resolved when DTT was added. Furthermore, monomeric Mcp3a migrated as a double band both under reducing and nonreducing conditions, which can most likely be attributed to incomplete unfolding in the sample loading buffer. *B*, enzymatic activity of legumain (2 nM) toward the AAN-AMC substrate measured in the presence or absence of 10 nM Mcp1a or Mcp3a using nonreducing or reducing (+DTT) assay buffer at pH 5.5. AAN-AMC, Z-Ala-Ala-Asn-7-amino-4-methylcoumarin; AEP, asparaginyl endopeptidase; Mcp3a, macrocypin 3a; Mcp1a, macrocypin 1a.

Even though the RCL sequences of different macrocypin isoforms were highly conserved in sequence alignments, we noticed that there were specific sequence variations at critical positions (Fig. 3*C*). While Mcp1a harbors a Ser75^Mcp^ in position P1′, Mcp3 harbors a cysteine at the equivalent position. Looking at the crystal structure of the legumain–Mcp1a complex, we observed that the P1′ side chain was in close proximity to the Sγ atom of the catalytic Cys189 residue on legumain (Fig. 2*B*). Based on this observation, we hypothesized that macrocypin 3a (Mcp3a) could potentially be a covalent-reversible legumain inhibitor, mediated by disulfide formation between the P1′-Cys75 of Mcp3a and Cys189 on legumain. To test this hypothesis, we recombinantly expressed Mcp3a, coincubated it with legumain at pH 4.0 to 7.0 and analyzed the reactions on SDS-PAGE using nonreducing and reducing sample loading buffer. Indeed, in the absence of DTT, we observed a band corresponding in size to the covalent legumain–Mcp3a complex (legumain: 36 kDa, Mcp3a: 20 kDa, legumain–Mcp3a complex: 56 kDa) (Fig. 4*A*). The band was resolved to AEP and Mcp3a bands, when DTT was added, which confirmed that the complex was stabilized by disulfide formation. Additionally, we observed that Mcp3a formed a covalent, disulfide mediated dimer, which was similarly resolved in the presence of DTT (Figs. 4*A*, S7, *A* and *B*). Furthermore, monomeric Mcp3a migrated as a double band on SDS-PAGE, most likely due to incomplete unfolding in the SDS-PAGE loading buffer (Fig. S4, *C* and *D*). Formation of the covalent AEP–Mcp3a complex was further confirmed using SEC experiments (Fig. S7*C*). The covalent complex eluted as a separate peak. SDS-PAGE analysis of the peak fractions showed that under nonreducing conditions, it contained a band at the expected height of the covalent complex, which was resolved when DTT was added to the SDS-PAGE sample loading buffer. Since covalent complex formation is typically associated with a long-lived interaction, we expected that Mcp3a would be a more potent legumain inhibitor than Mcp1a. Interestingly, when we compared the inhibition of legumain by Mcp1a and Mcp3a in the presence or absence of DTT, we observed similar inhibition for both inhibitors (Fig. 4*B*). In addition to the sequence variation in P1′ position, Mcp3 also harbors P3-Ile72Asp and P5-Phe70Ile variations relative to Mcp1a (Fig. 3*C*). Both residues are localized on the nonprime-binding side of the RCL. To test whether these additional sequence variations on Mcp3a cause additional differences in inhibition, we set up control reactions using an Mcp1a-S75C variant. This mutant only differs at P1′ position and therefore allows to specifically test the effect of the P1′-Cys on inhibition. Interestingly, the Mcp1a-S75C mutant showed an approximately 2-fold increase in inhibition compared to WT Mcp1a (Fig. S7*D*), suggesting that cysteine in P1′ position indeed strengthens the enzyme–inhibitor complex. This effect was however dampened in Mcp3a most likely due to additional, less favorable sequence variations in the P3 and P5 positions on the RCL.

In a next step, we were wondering whether P1′-Cys–mediated covalent complex formation could serve as a general strategy to increase the potency of other legumain inhibitors. To test this theory, we prepared a Clt2-T71C mutant, where Thr71^Clt^ at P1′ position was replaced by cysteine, incubated it with legumain, and subsequently analyzed the reactions by SDS-PAGE in the absence (Fig. S7*E*) or presence (Fig. S7*F*) of the reducing agent DTT. Indeed, in the absence of reducing agent, we observed a band at the expected height of the covalent legumain–Clt2-T71C complex (legumain: 36 kDa, Clt2-T71C: 18 kDa, legumain-Clt2-T71C: 54 kDa). This band was resolved when DTT was added to the samples. Similarly, Clt2-T71C was additionally prone to intramolecular disulfide formation, which led to the formation of a covalent dimer. The dimer migrated as a single band, when the samples were not heated prior to loading them on SDS-PAGE (Fig. S4*C*). When the samples were heated, the dimer converted to a double band, composed of (partly) folded and unfolded protein (Fig. S7*E*). Addition of DTT to the samples resolved the dimer bands to monomeric Clt2-T71C (Figs. S7*F* and S4*D*). Consistent with the observation of the covalent complex on SDS-PAGE, we also observed inhibition of legumain by the Clt2-T71C mutant using the fluorogenic Z-Ala-Ala-Asn-7-amino-4-methylcoumarin (AAN-AMC) substrate (Fig. S7*G*). Remarkably, the inhibitory potency of the P1′ cysteine variant Clt2-T71C was lower than that of the WT Clt2. P1′ cysteine-harboring mycocypins are partly present in a dimeric state and thereby engage the RCLs in the dimer interface. Consequently, the dimeric state is inhibitory inactive. Depending on the equilibrium of active monomer and inactive dimer states, the P1′ cysteine contribution to inhibition will strengthen or weaken the inhibition. In Clt2-T71C, the dimer band is much more dominant as in Mcp3a (Figs. 4*A* and S7*E*), explaining the weaker inhibition in Clt2-T71C. Unlike Mcp3a, disulfide-mediated covalent complex formation was most favorable at around pH 6.0 for the Clt2-T71C mutant (Fig. S7*E*). Importantly, for Mcp3a, Mcp1a-S75C, and Clt2-T71C, we observed covalent complex formation only with the intact, unprocessed inhibitor. In none of them, we observed a covalent complex composed of legumain and processed inhibitor (Figs. 4 and S7). Even more interestingly, we did not observe processing of Mcp3a by legumain, suggesting that covalent complex formation was restricted to the intact form of the inhibitor and that disulfide formation further prevented inhibitor processing, since the catalytic Cys189 on legumain was covalently blocked in the complex. Since Clt2 is, unlike Mcp1a, readily processed by legumain at pH 4.0 to 6.0 and since the potency of the Cys Sγ atom for disulfide formation is pH-dependent (pKa Cys Sγ ≈ 8.3 (34)), together this can explain why the covalent legumain–Clt2-T71C complex was mostly observed at around pH 6.0. In principle, pH 7.0 would favor disulfide formation even more than pH 6.0. However, at pH 7.0, legumain is prone to conformational destabilization (9).

To further investigate the relevance of the P1′ residue, we additionally prepared an Mcp1-S75A mutant and tested its inhibition of legumain. The S75A mutation showed a slight reduction in the inhibition of legumain by Mcp1a (Fig. S7*H*).

### The RCL interaction is not sufficient for inhibition

**Figure 5:**
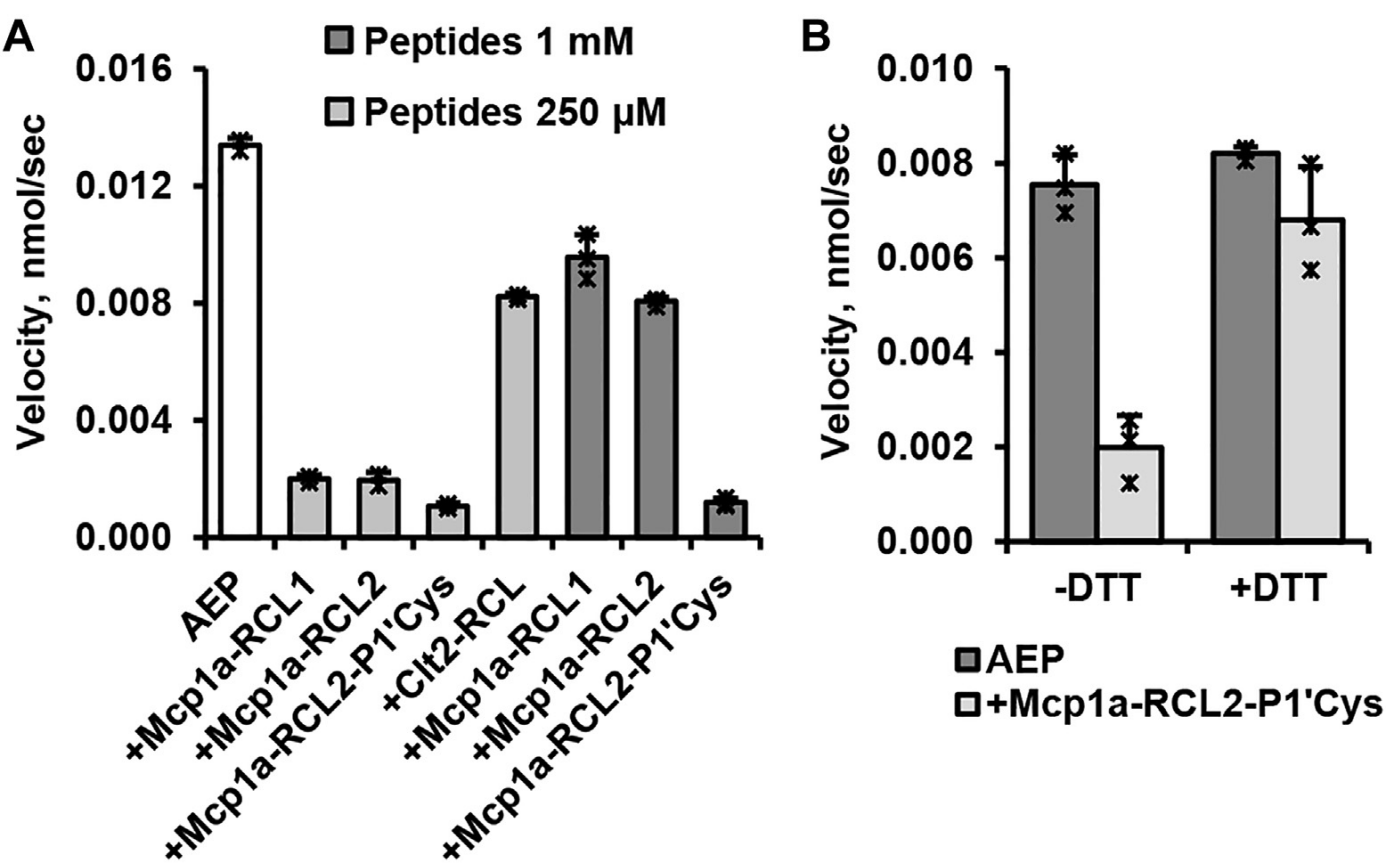
**RCL-derived peptides are inhibiting legumain activity.***A*, enzymatic activity of legumain toward the AAN-AMC substrate was measured at pH 5.5 in the presence or absence of 1 mM or 250 μM of the specified peptides. Clt2-RCL: Y^66^QGLNTP^72^, Mcp1a-RCL1: T^68^EFRIDNSIPGQ^79^, Mcp1a-RCL2: T^68^EFRIDNSIPGQ^79^G, Mcp1a-RCL2-P1′Cys: T^68^EFRIDNC^75^IPGQ^79^G. *B*, inhibition of legumain by the Mcp1a-RCL2-P1′Cys peptide is reduced in the presence of the reducing agent DTT, suggesting covalent, disulfide-mediated binding of Cys75 on the peptide to Cys189 on legumain. Peptide concentration: 250 μM. AAN-AMC, Z-Ala-Ala-Asn-7-amino-4-methylcoumarin; Clt2, clitocypin 2; Mcp1a, macrocypin 1a; RCL, reactive center loop.

Prompted by the observation that the RCL interaction is critical for inhibition, we were in a next step wondering whether the RCL sequences of Mcp1a and Clt2 would be sufficient for the inhibition of legumain. To answer this question, we designed a set of RCL-derived peptides. Specifically, we ordered a linear peptide derived from the Clt2 RCL sequence (Clt2-RCL: Y^66^QGLNTP^72^) and two head-to-tail cyclized peptides based on the RCL sequence of Mcp1a (Mcp1a-RCL1: T^68^EFRIDNSIPGQ^79^ and Mcp1a-RCL2: T^68^EFRIDNSIPGQ^79^G). Cyclization should further stabilize the electrostatic clamp on the Mcp1a-RCL peptides. Additionally, we designed a cyclic peptide based on the RCL sequence of Mcp1a but harboring a Ser to Cys variation at position P1’ (Mcp1a-RCL2-P1′Cys: T^68^EFRIDNC^75^IPGQ^79^G). Indeed, we observed inhibition of legumain by all four peptides (Fig. 5*A*). Interestingly, the cyclic Mcp1a-derived peptides bound with higher apparent affinity than the Clt2-derived peptide. This observation is in agreement with the generally higher affinity of Mcp1a to legumain than Clt2. And it is further highlighting the importance of the RCL conformation for the interaction with legumain. Importantly, the cyclic Mcp1a-RCL2-P1′Cys peptide showed strong legumain inhibition (∼20% residual activity) even at 250 μM concentration, where the WT peptide would still leave ∼80% residual activity. Furthermore, the interaction of the Mcp1-RCL2-P1′Cys peptide was sensitive to the presence of a reducing agent (Fig. 5*B*), providing evidence that the peptide was similarly establishing a covalent interaction to the catalytic Cys189 residue. However, overall, the affinity of all tested peptides was approximately three orders of magnitude lower (IC_50_ in the μM range) than the Mcp1a and Clt2 proteins (K_i_ Mcp1a: 2.18 ± 0.35 nM, K_i_ Clt2: 79 ± 3 nM; see Table 2), suggesting to us that the exosite interaction must be key for efficient inhibition.

### The exosite interaction defines the inhibitor as an inhibitor

**Figure 6:**
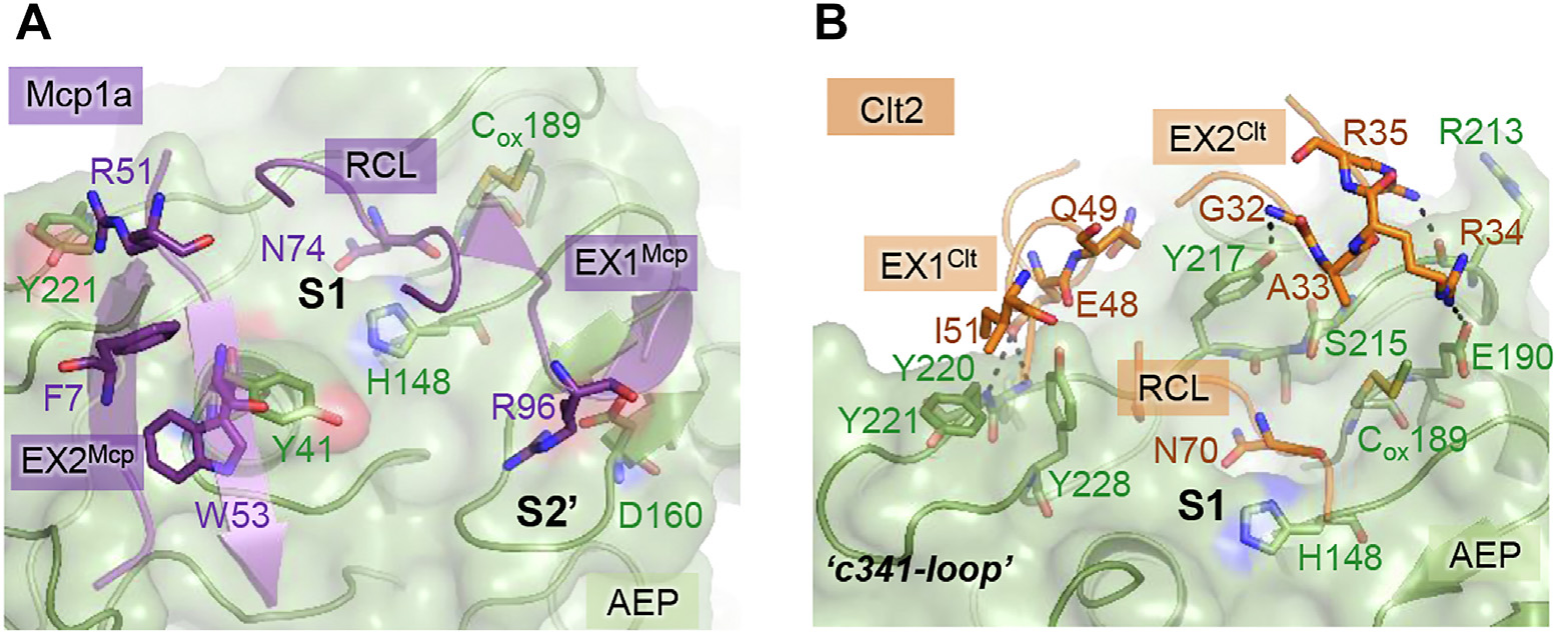
**Exosite interaction is essential for inhibition.***A*, view on the active site of legumain (*green*) bound to Mcp1a (*purple*). Exosite interactions are indicated as *sticks*. Additionally, the P1-Asn74 residue on the Mcp1a RCL is shown in *sticks*. *B*, view on the active site of legumain bound to Clt2 (*orange*). Exosite interactions are indicated as *orange sticks*. The P1-Asn70 residue on the Clt2-RCL is also indicated. Clt2, clitocypin 2; Mcp1a, macrocypin 1a; RCL, reactive center loop.

Closely analyzing the crystal structure of legumain in complex with Mcp1a, we found in addition to the RCL, two exosites that mediate binding to legumain (Figs. 1, 6*A* and S8*A*). Exosite 1 (EX1^Mcp^) is built up by a conserved Arg96^Mcp^ residue on Mcp1a, which is establishing ionic interactions to Asp160 on legumain. Asp160 forms the eastern wall of the S2′ substrate-binding site on legumain. Thereby, EX1^Mcp^ is mediating prime-side interactions and is extending the substrate-like interaction of the macrocypin RCL. Additionally, we identified Arg51^Mcp^ which is hydrogen bonding to Tyr221 on the legumain S4/5 site and thereby also extending interactions to the nonprime substrate-binding site. Exosite 2 (EX2^Mcp^) is formed by Phe7^Mcp^ and Trp53^Mcp^, which mediate hydrophobic interactions to Tyr41 in the south of the nonprime substrate-binding cleft of legumain. While EX1^Mcp^ is localized to the crown of Mcp1a, EX2^Mcp^ is localized to the trunk of the inhibitor (Fig. 1*F*). Since both exosites are sequentially distant to the RCL, they could potentially stabilize the enzyme–inhibitor complex, also after processing after the P1-Asn74^Mcp^ residue by legumain. To test this hypothesis, we prepared an EX1^Mcp^ Mcp1a-R96A mutant and tested its interaction with legumain. Indeed, the EX1^Mcp^ mutant showed an approximately 100-fold reduction in legumain inhibition (Table 2). Additionally, we found that the Mcp1a-R96A mutation led to an increase in inhibitor processing at pH 4.0 to 6.0 (Fig. S8*B*), which suggested that if the exosite interaction was removed, Mcp1a was rather a substrate to legumain than an inhibitor. In a previous study, we observed a similar exosite interaction also in cystatins (33). Exosite interaction in that case significantly improved the pH-stability of legumain within the complex. This effect could be attributed to the shielding of the electrostatic stability switch, which is an area dense in negative charge that is localized south of the active site and on the prime substrate-binding sites. Thermal shift assays showed that Mcp1a has a similar pH-stabilizing effect on legumain at pH 6.0 (Fig. S8*C*). This effect was abolished when the EX1^Mcp^ Mcp1a-R96A variant was coincubated with legumain, which further confirmed the importance of this residue for the interaction with legumain. In line with this observation, the Mcp1a-RCL mutants (Mcp1a-D73A and Mcp1a-R71E), where the RCL conformation was disturbed, were similarly unable to stabilize legumain at near neutral pH (Fig. S6*E*).

In stark contrast to Mcp1a, the Clt2 exosites are mediating interactions to the nonprime substrate-binding sites and the north of the nonprime substrate-binding cleft on legumain (Figs. 1, 6*B* and S8*F*). Specifically, exosite 1 (EX1^Clt^) interactions are mediated by residues Glu48^Clt^, Gln49^Clt^, and Ile51^Clt^ on Clt2, which are interacting by hydrogen bonding and hydrophobic interactions with the c341-loop on legumain. The c341-loop serves as a template for nonprime substrate binding. EX1^Clt^ interaction involves Tyr220, Tyr221, and Tyr228 on legumain. Exosite 2 (EX2^Clt^) is mediating interactions to the north of the nonprime substrate-binding cleft *via* residues Gly32^Clt^–Arg35^Clt^. Similarly, EX2^Clt^ is establishing interactions to the c341-loop *via* residues Arg213, Ser215, and Tyr217 on legumain. Both exosites are localized to the crown of the inhibitor (Fig. 1*F*). To test the relevance of these residues for the interaction with legumain, we prepared an EX1^Clt^ Clt2-E48A-Q49A-I51A triple mutant (Clt2-3x) which should disrupt the interaction to the S4/5 site on legumain. As expected, the triple mutant showed reduced inhibition of legumain (Table 2) and an increase in hydrolysis by legumain (Fig. S8*D*). This finding clearly showed that similar to the Mcp1a-R96A mutant, the Clt2-3x mutant behaved more like a substrate to legumain than an inhibitor. Thereby we could confirm that the Clt2 exosite interaction is indeed important for the inhibition of legumain. Interestingly and unlike Mcp1a, Clt2 did not have a significant pH-stabilizing effect on legumain in a thermal shift assay (Fig. S8*E*). This observation further confirmed that the pH-stabilizing effect was directly linked to the electrostatic stability switch, which is localized mainly to the prime substrate-binding sites and south of the substrate-binding cleft on legumain, which is addressed by Mcp1a but not by Clt2. Together, these results clearly show that it is the exosite interaction that turns the mycocypins into inhibitors rather than substrates.

### Mycocypins inhibit specific legumain activities with different affinity

**Figure 7:**
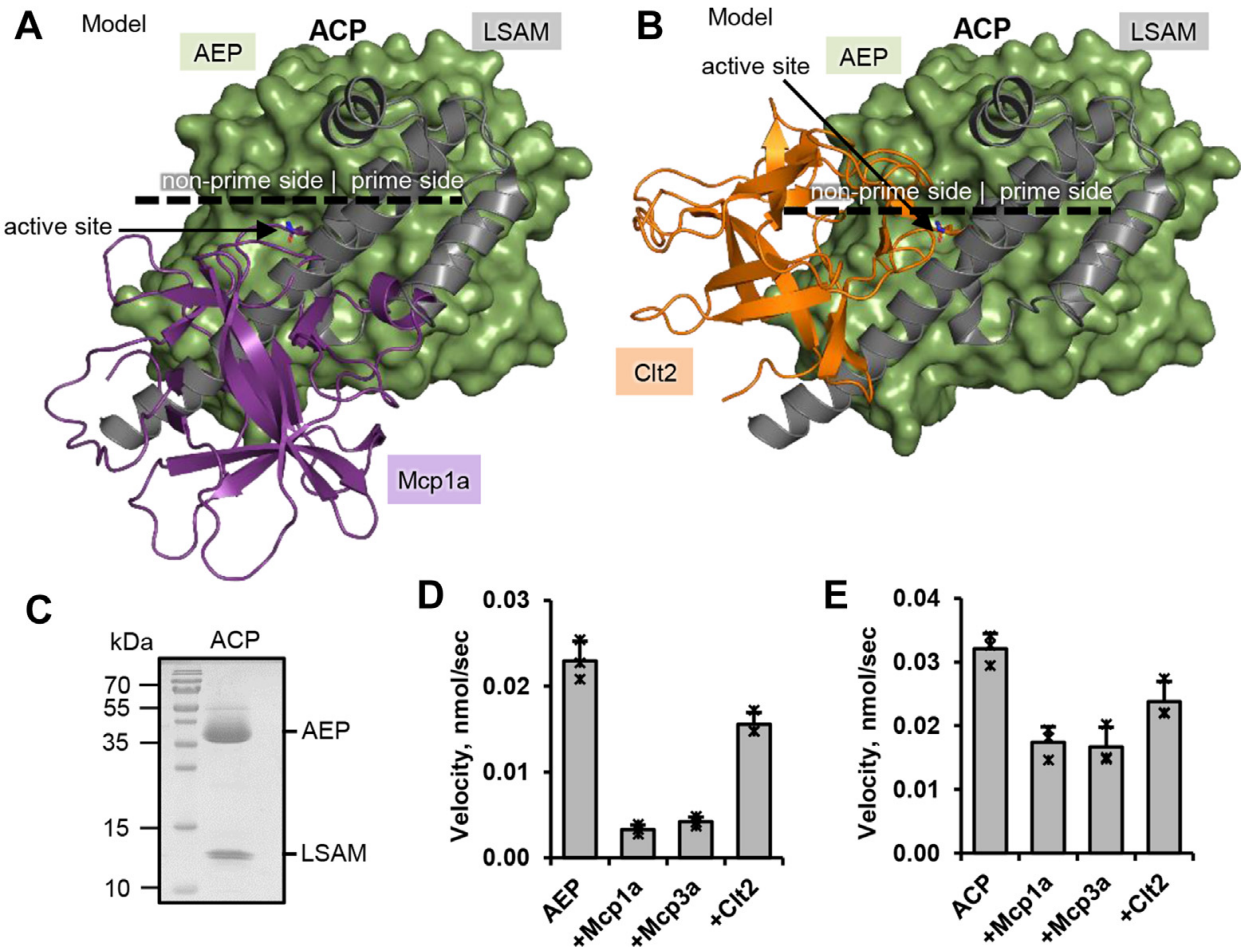
**Mycocypins inhibit the AEP and ACP forms of legumain with different efficiency.***A*, model of an ACP–Mcp1a complex based on the crystal structures of human prolegumain (pdb 4fgu) and the AEP–Mcp1a complex. ACP: Asparaginyl CarboxyPeptidase, consisting of the catalytic AEP domain and the LSAM domain. *B*, model of an ACP–Clt2 complex based on crystal structures of human prolegumain and the AEP–Clt2 complex. *C*, SDS-PAGE showing the ACP form of legumain. *D*, enzymatic activity of fully activated human legumain (AEP, catalytic domain only, 2 nM) measured as turnover of the AAN-AMC substrate at pH 5.5, in the presence or absence of 10 nM Mcp1a, Mcp3a, or Clt2. *E*, same as (D) but using the ACP (2 nM) form of legumain. AAN-AMC, Z-Ala-Ala-Asn-7-amino-4-methylcoumarin; ACP, asparaginyl carboxypeptidase; AEP, asparaginyl endopeptidase; Clt2, clitocypin 2; LSAM, legumain stabilization and activity modulation domain; Mcp1a, macrocypin 1a; Mcp3a, macrocypin 3a.

Depending on the pH-environment, prolegumain will activate to specific AEP and asparaginyl carboxypeptidase (ACP) forms. AEP is generated upon incubation of prolegumain at pH ≤ 4.0, which results in the complete autocatalytic removal of the activation peptide and the LSAM domain. ACP is generated upon incubation of prolegumain at pH > 4.0 to ≤ 5.0. Under these conditions, the activation peptide is removed but the LSAM domain remains electrostatically bound to the catalytic AEP domain (9). Importantly, AEP and ACP differ in their substrate specificity. While in AEP, the substrate-binding cleft is fully accessible both on the prime and nonprime sides, whereas in ACP, only the nonprime and P1′ substrate-binding sites are accessible. These differences in active site accessibility led us to the hypothesis that the ACP and the AEP forms of legumain would show differences in inhibition by the mycocypins. To test this hypothesis, first, we prepared models of ACP in complex with Mcp1a or Clt2, based on the crystal structures of human prolegumain (pdb 4fgu) and the AEP–Mcp1a and AEP–Clt2 complexes (Fig. 7, *A* and *B*). These models suggested that the binding mode exploited by Clt2 is compatible with binding both to AEP and ACP. We did not observe major sterical clashes between Clt2 and the LSAM domain within this model (Fig. 7*B*). However, we did observe clashes between the LSAM domain and Mcp1a in the model of the ACP–Mcp1a complex (Fig. 7*A*), which suggested that Clt2 would inhibit AEP and ACP equally well, but Mcp1a would be a better inhibitor of AEP than ACP. To test this assumption, we tested the inhibition of AEP and ACP (Fig. 7*C*) by Mcp1a, Mcp3a, and Clt2 using the AAN-AMC fluorescence substrate (Fig. 7, *D* and *E*). In line with our hypothesis, we found that Clt2 was inhibiting AEP and ACP equally well, leading to a residual activity of approximately 60% – 70% (Fig. 7, *D* and *E*). Importantly, Mcp1a and Mcp3a were inhibiting AEP approximately three times better than ACP, consistent with our finding that macrocypins need to establish prime side exosite interactions for inhibition, which are sterically not easily accessible in ACP.

### Mycocypins inhibit different legumain isoforms with specific affinities

**Figure 8:**
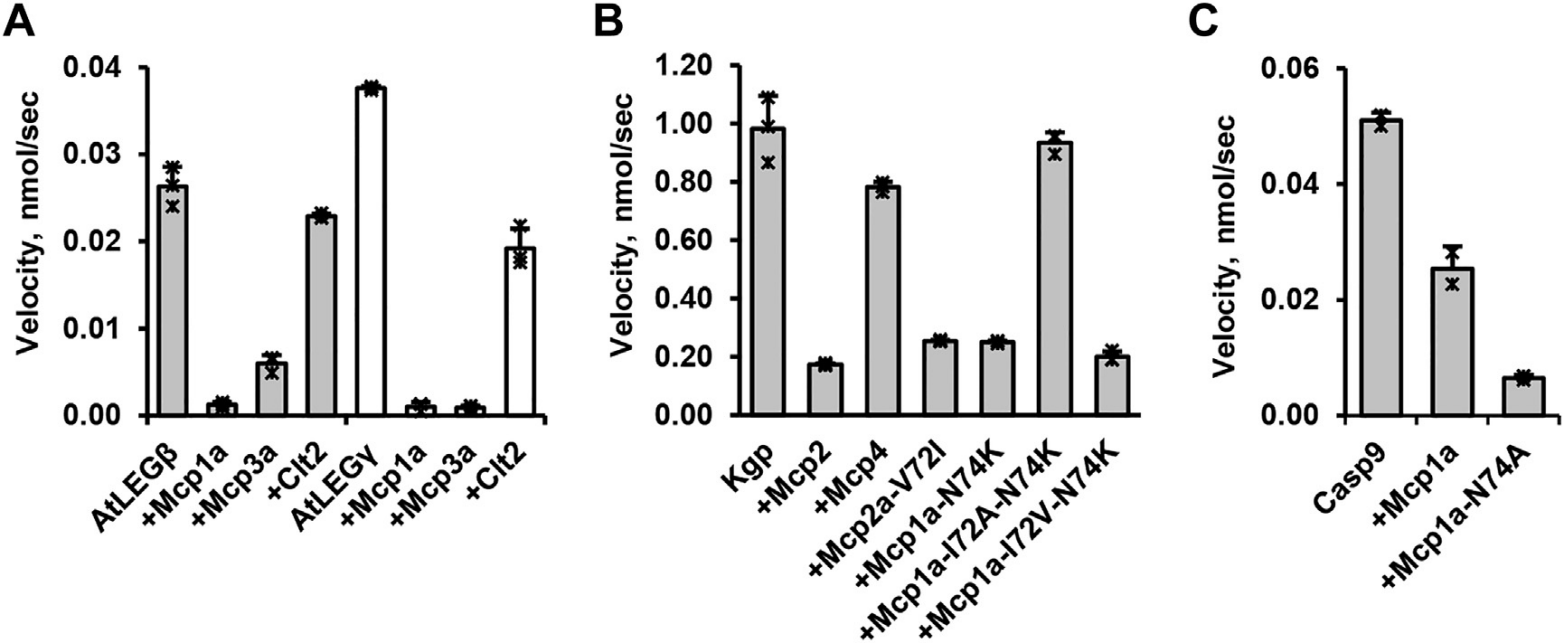
**Mycocypins inhibit different legumain isoforms and clan CD proteases with different efficiency.***A*, inhibition of *Arabidopsis thaliana* legumain isoforms β (AtLEGβ, *grey bars*) or γ (AtLEGγ, *white bars*) (50 nM) by indicated mycocypins at 250 nM concentration was measured as turnover of the AAN-AMC substrate at pH 5.5. *B*, enzymatic activity of *Porphyromonas gingivalis* gingipain K (Kgp) measured as turnover of the Tos-Gly-Pro-Lys-pNA substrate after incubation with indicated mycocypin variants at 5 μM concentration. *C*, enzymatic activity of ΔCARD-caspase-9 (1 μM) measured as turnover of the VAD-AMC substrate in the presence of 25 μM of Mcp1a or Mcp1a-N74A. AAN-AMC, Z-Ala-Ala-Asn-7-amino-4-methylcoumarin; Clt2, clitocypin 2; Mcp1a, macrocypin 1a; VAD-AMC, Z-Val-Ala-Asp-7-amino-4-methylcoumarin.

When we analyzed sequences of legumain inhibitory mycocypins, we observed specific variations of the RCL but also of the exosite sequences. Based on this observation, we were wondering whether such isoform-specific sequence variations might encode for specific inhibitory activities toward specific legumain isoforms. To test this hypothesis, we compared the inhibitory potential of Mcp1a, Mcp3a, and Clt2 toward human legumain and *Arabidopsis thaliana* legumain isoforms β and γ (AtLEGβ, AtLEGγ). Overall, all three investigated legumain variants were inhibited by the tested mycocypins (Figs. 7*A* and 8*A*). Interestingly, however, all of them were most potently inhibited by the macrocypins and less potent by Clt2, in agreement also with previously published data (27). Furthermore, macrocypins 1a and 3a showed more potent inhibition of AtLEGγ than AtLEGβ. And, while human legumain and AtLEGγ were similarly well inhibited by Mcp1a and Mcp3a, AtLEGβ showed an approximately four-fold reduced inhibition by Mcp3a as compared to Mcp1a. To get a better understanding of these differences in inhibition, we prepared models of AtLEGβ and AtLEGγ in complex with Mcp1a or Clt2, based on the crystal structure of human legumain in complex with Mcp1a or Clt2. The models showed that the prime side EX1^Mcp^ interaction mediated by Arg96^Mcp^ on Mcp1a was not possible in the investigated plant legumains, since the corresponding interaction partner Asp160 on human legumain is replaced by His182 in AtLEGβ and Tyr190 in AtLEGγ (Fig. S9*A*). Both residues are more bulky and do not allow ionic interactions to EX1^Mcp^ Arg96^Mcp^. Additionally, His182 on AtLEGβ even has an opposing charge, which will cause electrostatic repulsion of EX1^Mcp^. Looking at EX2^Mcp^, we found that Tyr41 on human legumain is replaced by Gly63 in AtLEGβ and Trp71 in AtLEGγ. While Trp71 is still capable of mediating hydrophobic interactions to Trp53^Mcp^, Gly63 is not. Together, these differences indicated that the interaction of Mcp1a to the southern rim on AtLEGβ is weaker, which can explain why Mcp’s are better inhibitors of AtLEGγ than AtLEGβ. Additionally, we found that the cyclic protein recognition motif insertion on the nonprime substrate-binding cleft of AtLEGβ and γ is in steric conflict with EX1^Clt^ on Clt2 (Fig. S9*B*), which can provide an explanation why Clt2 is an even worse inhibitor of plant legumains than human legumain. Together, these results clearly show that mycocypins will inhibit different legumain variants with different affinities.

### Mycocypins also inhibit other clan CD proteases

When we were looking at the RCL sequences of so far identified mycocypin sequences, we noticed variations not only at the P5, P3, and P1′ positions but also at the P1 position. Interestingly, Mcp2 and Mcp4 sequences showed a lysine residue at P1 position, which suggested that they might be inhibiting other clan CD proteases with specificity for lysine. Potential candidates included paracaspases and the bacterial *Porphyromonas Gingivalis* gingipain K (Kgp) (35, 36). To test the relevance of Mcp2 and Mcp4 as inhibitors of clan CD proteases, we recombinantly expressed macrocypin 2a (Mcp2a) and macrocypin 4a (Mcp4a) and tested their reactivity toward Kgp as a model enzyme. Interestingly, we found that both variants were inhibiting Kgp activity, with different affinities though (Fig. 8*B*). While we determined a K_i_ of 1.47 ± 0.04 μM for Mcp4a, Mcp2a showed a 10-fold increase in affinity with a K_i_ of 0.18 ± 0.02 μM. Even though both Mcp variants showed only minor differences in the RCL sequences, they showed significant differences in inhibition. Analyzing the sequence variations more closely, we found that Mcp2a and Mcp4a were differing at position P3, which is a valine in Mcp2a and alanine in Mcp4a (Fig. 3*D*). To understand the differences in affinity, we prepared a model of a Kgp–Mcp2a complex by superposing the crystal structure of legumain in complex with Mcp1a onto the crystal structure of Kgp (Fig. S10*A*). The model suggested that a P3-Val (Mcp2) or P3-Ile residue (Mcp1a) would fit better into the Kgp substrate-binding cleft than a P3-Ala, as found in Mcp4. To test the relevance of the P3 residue for the inhibition of Kgp, we prepared Mcp1a-I72A-N74K, Mcp1a-I72V-N74K, and Mcp2a-V72I mutants. Indeed, we found that mutation of Ile72^Mcp^ to Ala had a negative effect on the propensity of Mcp1a-N74K to inhibit Kgp (Fig. 8*B*). Mutations of P3-Ile to P3-Val or vice versa had no negative impact on inhibition. Using mass spectrometry experiments, we could furthermore show that Mcp1a-N74K was cleaved by Kgp after the P1-Lys74 residue, confirming a similar, substrate-like binding mode. Additionally, introducing a cysteine residue at the P1′ position of Mcp2a (Mcp2a-S75C) had a positive effect on the inhibition of Kgp, suggesting a similar covalent bond formation with the catalytic cysteine residue (Fig. S10*B*).

Further analysis of the macrocypin RCL sequences revealed a conserved aspartic acid residue in position P2. Knowing that caspases specifically cleave substrates after aspartic acid residues, we were in a next step wondering if macrocypins would also be suited as caspase inhibitors. To test the propensity of macrocypins as caspase inhibitors, we recombinantly expressed active caspase-9 and used it as a test enzyme. Interestingly, WT Mcp1a showed only little inhibition of caspase-9 (Fig. 8*C*). Since caspases have a strong preference for small residues at position P1′, we prepared a Mcp1a-N74A mutant to further test the relevance of the P1′ position. Indeed, the Mcp1a-N74A variant showed inhibition of caspase-9, which confirmed that the P2-Asp73 on the Mcp1a RCL may serve as a P1 residue for the inhibition of caspases.

## Discussion

**Figure 9:**
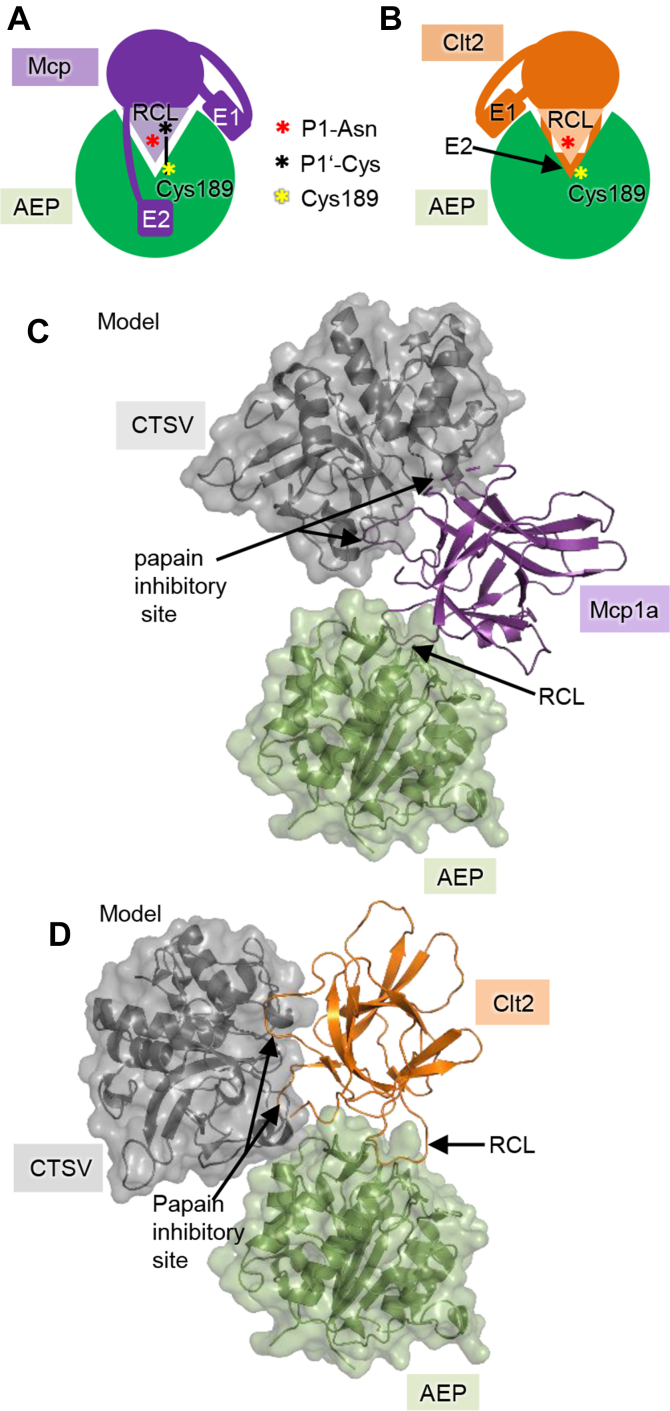
**Macrocypins and clitocypins interact differently with legumain.***A*, Mcp’s (*purple*) interact with legumain (*green*) *via* their reactive center loop (RCL) and two exosites (E1 and E2) that stabilize the complex by binding to the prime substrate-binding sites and the area south of the active site. Additionally, Mcp3a harbors a cysteine at position 75 on the RCL (P1′), which can mediate covalent complex formation by disulfide linkage to the catalytic Cys189 on legumain. *B*, similarly, Clt’s (*orange*) also use an RCL and two exosites to inhibit legumain. However, different to Mcp’s, Clt’s exosite is mediating interactions to the nonprime substrate-binding sites (E1) and to the area in the north of the active site (E2, behind the active site). *C*, model of a ternary complex composed of Mcp1a (*purple*) bound to legumain (*green*) and cathepsin V (CTSV, *gray*). *D*, model of a ternary complex composed of Clt2 (*orange*), legumain, and CTSV. Models were prepared by superposing the structures of Mcp1a or Clt2 in complex with legumain onto the crystal structures of cathepsin V in complex with a clitocypin (pdb 3h6s). Clt2, clitocypin 2; Mcp1a, macrocypin 1a; Mcp3a, macrocypin 3a.

Even though Clt2 and Mcp1a only share a low sequence identity of 19%, their overall fold and their biochemical, and biophysical properties are highly similar. Our structural and functional analysis of legumain in complex with Clt2 and Mcp1a showed that both inhibitors harbor a RCL with a conserved asparagine residue that is critical for the inhibition of legumain (Fig. 9*A*). In both inhibitors, the RCL binds to the legumain active site in a substrate-like manner, with the asparagine residue functioning as P1-residue. By this binding mode, both Clt’s and Mcp’s represent legumain substrates. Indeed, even though the RCL–protease interaction is necessary for mycocypin recognition, it is not sufficient for inhibition. Instead, both inhibitors additionally encode exosite interactions, which are required to stabilize the enzyme–substrate complex and turn the substrates into inhibitors. Importantly, the exosite interactions encoded by clitocypins and macrocypins are localized to different regions on the inhibitors, even though their overall fold is highly similar. Clitocypin 2 utilizes loops on the crown of the tree to interact with the nonprime substrate-binding side and the area north to the active site of legumain. Mcp1a utilizes amino acids localized to the crown and the trunk to interact with the prime-side and the area south to the active site of legumain (Fig. 9*A*). Taken together, our structural and functional data suggest a two-step mode of inhibition. In a first step, the inhibitors are recognized like substrates, utilizing the P1-Asn residue on the RCL. In parallel, substrate recognition goes along with binding of the exosites to legumain, thereby stabilizing the enzyme–inhibitor complex. A second, regulatory step occurs only once the inhibitor is stably and productively bound to the enzyme. In this step, pH- and conformation-dependent cleavage after the P1-Asn residue on the RCL may occur, which goes along with a pH-dependent increase in affinity in Clt2. While the affinity of legumain toward processed Clt2 remained similar to intact inhibitor at pH 6.0, it increased approximately three fold at pH 4.0. This increase in affinity may be partly attributed to protonation of residues like His148, which will be positively charged at pH 4.0 and might form a salt bridge with the neo C-terminus of the cleaved inhibitor or to a pH-dependent increase in flexibility of the cleavage products. Furthermore, the regulatory phase may also include the formation of a covalent complex, if the inhibitor harbors a cysteine in P1′ position. Disulfide formation may be mediated by a P1′-Cys on the mycocypin and the catalytic Cys189 on legumain. Importantly, disulfide formation is in general unfavorable at acidic pH due to protonation of the Cys Sγ (pKa ≈ 8.3 (34)). However, complex formation leads to a local increase in concentration and thereby enforces proximity of the two cysteine residues, which allows them to form the disulfide even though pH is unfavorable. This finding opens up a new strategy for drug development. Specifically, small molecule and peptide-based inhibitors could be designed that bind to legumain with even higher affinity due to covalent binding to the legumain active site. We could already provide a proof of this concept using cyclic Mcp1a-RCL–derived peptides harboring Cys in P1′ position. Furthermore, we could show that differences in affinity between clitocypins and macrocypins toward legumain can be explained by their RCL conformation and mode of exosite interaction. In general, macrocypins are more potent inhibitors of legumain than clitocypins. This is likely due to intramolecular stabilization of the RCL conformation and their different exosite interaction, which additionally encodes a pH-stabilizing effect on legumain.

A mechanistically similar two-step mode of inhibition also evolved in the family 2 cystatins, even though they structurally belong to a completely different protein family. Cystatins harbor a substrate-like RCL that serves as a bait and an exosite that is stabilizing the enzyme–substrate complex and turns the substrate into an inhibitor. The mechanistic similarity suggests that this mode of inhibition may serve as a universal strategy to target legumain. Interestingly, even though macrocypins and clitocypins similarly show a substrate-like processing of the RCL by legumain, unlike cystatins, we did not observe ligation of the cleaved inhibitors. This may indicate that the newly generated N-terminus of the primed cleavage product is flexible and quickly released from the active site, which consequently prevents religation of the RCL. Even though the RCL is in principle internally stabilized in Mcp’s, the newly generated N-terminus may still be flexible after cleavage. When we superposed the crystal structures of legumain with cystatin E (pdb 4n6o), Clt2, and Mcp1a, we found that the scissile peptide bond was slightly shifted in Mcp1a and significantly shifted in Clt2, relative to cystatin E (Fig. S11), which indicates that the detailed geometry of the scissile peptide bond may also be critical for ligation to work. Furthermore, in the crystal structure of the AEP–Clt2 complex, we also found two ordered water molecules in close proximity to the scissile peptide bond and the catalytic cysteine residue. These water molecules may additionally disfavor religation but rather mediate hydrolysis. Analyzing the structure of the AEP–Mcp1a complex, we did not observe a structured water molecule. However, since the overall B-factor of this structure was especially high (93.6 Å^2^), only few ordered water molecules are to be expected.

We furthermore found that while Clt2 was inhibiting the AEP and ACP forms of legumain equally well, macrocypins were better inhibitors of AEP than ACP. This observation is in line with the steric occlusion of the prime substrate-binding sites in ACP by the LSAM domain. However, since the macrocypins were still capable of inhibiting ACP, they may cause a competitive displacement of the LSAM domain. It is tempting to speculate that macrocypins and clitocypins might have evolved as AEP and ACP inhibitors, respectively. Our studies furthermore showed that macrocypins and clitocypins can in principle inhibit different legumain family members from different organisms with different affinities, which suggests that they may have evolved to address different legumain isoforms. Modelling suggests that Clt2 is primarily directed toward human-type legumain’s, which are lacking the cyclic protein recognition motif insertion. Modeling further suggests that macrocypins and clitocypins can potentially bind cathepsins and legumain simultaneously (Fig. 9, *C* and *D*), which implies that they are bispecific, janus-faced inhibitors. Interestingly, EX1^Clt^ on Clt2 is sequentially and sterically close to the papain-binding site. However, the two sites evolved such, that they are not overlapping each other. Consequently, mushrooms might use mycocypins to defend themselves against predators, which utilize papain-like enzymes and legumains as digestive enzymes as, for example, found in schistosome or tick (37, 38). Furthermore, we provide evidence that macrocypins can also be utilized to inhibit other clan CD proteases like the bacterial gingipains. Kgp is a lysine-specific cysteine protease and is the major virulence factor of the pathogen *P. gingivalis* because it is cleaving various extracellular matrix proteins of the host (35, 36). The propensity of Mcp2a to inhibit Kgp might be further exploited *in vitro* for the development of new protein or peptidic Kgp inhibitors. Furthermore, mycocypins may also provide a valuable scaffold for the development of inhibitors directed toward other clan CD proteases.

Taken together, we could decipher the inhibitory function of mycocypins toward legumain, which uncovered new aspects of its activity regulation. This knowledge may be translated to the design of selective, stable legumain or clan CD protease inhibitors, which may provide a new framework, for example, for target validation in drug design. In this study, we provide first evidence for the inhibitory potential of cyclic, RCL-based inhibitors harboring a cysteine in position P1’.

## Experimental procedures

### Cloning

*M. procera* Mcp1a (B9V973), Mcp2a (B9V977), Mcp3a (B9V979), Mcp4a (B9V982), and *C. nebularis* Clt2 (Q3Y9I4) full-length cDNA sequences were purchased from Eurofins Genomics.

Expression constructs were subcloned into the pET-22b(+) expression vector using NdeI and XhoI restriction enzyme cleavage sites, which were added directly 5′ or 3′ of the cDNA sequence. The expression constructs contained a C-terminal His_6_-tag for Ni^2+^-affinity purification. Various point mutations were introduced using round the horn mutagenesis, which is based on the inverse PCR method, as described earlier (39, 40). Correctness of expression constructs was confirmed *via* DNA-sequencing by Eurofins Genomics.

### Protein expression and purification

Recombinant expression, purification, and auto-activation of human prolegumain were done as previously described^2^. Briefly, the human prolegumain expression construct, which harbored a C-terminal His_6_-tag for purification, was stably transfected into the LEXSY P10 host (*Leishmania tarentolae* expression system, Jena Bioscience). Positive clones were selected using nourseothricin (Jena Bioscience). Cells were grown in brain heart infusion medium (Carl Roth) containing Hemin (Applichem), Pen-Strep (Penicillin-Streptomycin, Carl Roth), and nourseothricin, and incubated shaking (140 rpm) at 26 °C until an A_600_ ∼3 was reached. Cells were pelleted *via* centrifugation, and His_6_-tagged prolegumain was harvested from the supernatant by batch incubation with Ni^2+^-NTA Superflow resin (Qiagen). Following Ni^2+^-purification, elutions were concentrated using Amicon Ultra centrifugal filter units (MWCO: 10 kDa; Cytiva) and buffer exchanged *via* PD-10 columns (Cytiva) to get the protein in the final buffer 20 mM Tris pH 7.0, 50 mM NaCl, and 2 mM DTT. For subsequent assays, prolegumain was activated to the AEP at pH 3.5 as described previously. Activation to the ACP form was achieved upon incubation of prolegumain at pH 4.5. Progress of autocatalytic activation was monitored on SDS-PAGE. Subsequently, the activated proteins were concentrated using Amicon Ultra centrifugal filter units (MWCO: 10 kDa, Cytiva) and further purified using SEC utilizing the Äkta FPLC system equipped with a Superdex 75 10/300 Gl column (Cytiva) equilibrated in a buffer composed of 20 mM citric acid pH 4.0, 50 mM NaCl, and 2 mM DTT in case of AEP and 20 mM citric acid pH 5.0 and 50 mM NaCl in case of ACP. Similarly, *A. thaliana* prolegumain isoforms β and γ were expressed in LEXSY and purified as described previously (39, 41).

Mycocypins were expressed in *E. coli* BL21(DE3) cells. Expression plasmids were chemically transformed into the *E. coli* cells and positive clones were selected using ampicillin. Expression cultures were grown in LB medium, supplemented with 100 μg/ml ampicillin, at 37 °C and shaking at 220 rpm, until an A_600_ ∼ 0.8 was reached. Subsequently, cultures were transferred to 25 °C and expression was induced upon addition of 1 mM IPTG. Following overnight incubation, cells were harvested by centrifugation (4000 rpm, 10 min, 4 °C), and the cell pellet was resuspended in a buffer composed of 50 mM Tris pH 7.5 and 300 mM NaCl (Mcp2a, 3a, 4a, and Clt2) or 50 mM Tris pH 7.5 and 2 mM EDTA (Mcp1a). Subsequently, cells were lysed by sonication on ice at 40% power, 4 times for 45 s. The lysed cells were centrifuged at 17,500*g* for 30 min at 4 °C to separate the soluble and insoluble fractions. While Clt2, Mcp2a, and Mcp4a were expressed as soluble forms, Mcp1a was expressed as nonclassical inclusion bodies and Mcp3a was expressed in soluble form and as nonclassical inclusion bodies. For Mcp1a, the pellet containing the nonclassical inclusion bodies was solubilized in a buffer composed of 50 mM Tris pH 7.5, 2 mM EDTA, and 3 M urea, for 2 to 4 h at 4 °C (25). Subsequently, the sample was centrifuged at 17,500*g* for 30 min and 4 °C, to separate the soluble from the insoluble fraction. The supernatant was transferred into a dialysis tubing (Spectrum MWCO 3500 Da) and placed in 2 l dialysis buffer (50 mM Tris pH 7.5 and 300 mM NaCl) overnight at 4 °C. On the next day, the tube was transferred into 2 l of fresh dialysis buffer and stirred at room temperature (21 °C) for another 2 h, then centrifuged at 17,500*g* for 30 min and 4 °C. The soluble fraction containing correctly folded Mcp1a was further subjected to Ni^2+^-affinity purification and protein was eluted using a buffer composed of 50 mM Tris pH 7.5, 300 mM NaCl, and 250 mM imidazole. The elutions were concentrated using Amicon Ultra centrifugal filter units (MWCO: 3 kDa, Cytiva) and subjected to SEC utilizing the Äkta FPLC system equipped with a Superdex 75 10/300 Gl column (Cytiva) preequilibrated in buffer composed of 20 mM Tris pH 7.5 and 50 mM NaCl. Clt2, Mcp2a, Mcp3a, and Mcp4a, which were found in the soluble fraction after cell lysis, were directly subjected to Ni^2+^-purification and SEC, as described for Mcp1a.

ΔCARD-caspase-9 was expressed and purified as described previously (42).

### Peptides

Peptides derived from the RCL sequences of Mcp1a (Mcp1a-RCL1: T^68^EFRIDNSIPGQ^79^, Mcp1a-RCL2: T^68^EFRIDNSIPGQ^79^G, and Mcp1a-RCL2-P1′Cys: T^68^EFRIDNC^75^IPGQ^79^G) and Clt2 (Clt2-RCL: Y^66^QGLNTP^72^) were purchased from JPT Peptide Technologies and dissolved in DMSO to a final concentration of 100 mM.

### Enzymatic activity assays

Legumain protease activity was monitored using the fluorogenic peptide substrate Z-Ala-Ala-Ans-AMC (AAN-AMC, Bachem) at 460 nm after excitation at 380 nm in an Infinite M200 Plate Reader (Tecan) at 37 °C. The assay buffer contained 50 mM citric acid pH 5.5, 100 mM NaCl, 0.05% Tween-20, and 50 μM Z-AAN-AMC substrate. The reaction was started by the addition of 2 nM legumain. Inhibition of legumain was tested using assay buffer supplemented with 10 nM macrocypins, 2 μM Clt2, or 1 mM or 250 μM RCL peptide, respectively. If indicated, the assay buffer was additionally supplemented with 2 mM of the reducing agent DTT. To test the inhibition of *A. thaliana* legumain isoforms β and γ, we used assay buffer containing 2 mM DTT and 250 nM Mcp1a, Mcp3a, or Clt2, respectively. The reaction was started by the addition of 250 nM of the respective enzyme. Activity of caspase-9 was assayed in a buffer composed of 0.5 M Na_3_citrate, 50 mM Hepes pH 7.5, 100 mM NaCl, 0.05% Tween-20 and 100 μM of the fluorogenic Z-Val-Ala-Asp-AMC (Bachem) substrate. Activity was measured at 460 nm after excitation at 380 nm in an Infinite M200 Plate Reader (Tecan) at 37 °C and an enzyme concentration of 1 μM. Inhibition of caspase-9 by Mcp1a and Mcp1a-N74A was tested after supplementing the assay buffer with 25 μM of the respective inhibitor. Full-length *P. gingivalis* Kgp was kindly provided by Prof. Jan Potempa. Kgp activity was assayed in a buffer composed of 200 mM Tris pH 7.5, 150 mM NaCl, 5 mM CaCl_2_, 0.03% NaN3, and 4 mM L-cysteine hydrochloride monohydrate supplemented with 830 μM of the chromogenic substrate Tos-Gly-Pro-Lys-pNA (Bachem). The reaction was started by the addition of 0.25 μM enzyme (final concentration). Subsequently, the increase in absorbance at 405 nm was measured at 37 °C. To test the inhibition of Kgp by Mcp1a, Mcp2a, Mcp4a, Mcp1a-N74K, Mcp1a-I72A-N74K, Mcp1a-I72V-N74, and Mcp2a-V72I, the enzyme was preincubated with the respective inhibitor in assay buffer lacking the chromogenic substrate, for 10 min at 25 °C. Subsequently, activity was tested at 0.2 μM enzyme and 5 μM inhibitor concentration. For all activity measurements, activity was calculated as velocity in nmole per second, if not specified differently. For K_i_ determination, Kgp was preincubated with increasing concentrations of Mcp2a or Mcp4a in assay buffer lacking the chromogenic substrate, for 10 min at 25 °C. Subsequently, the enzyme-inhibitor mix was added to the reaction buffer containing the substrate and activity was measured as an increase in absorption at 405 nm for 10 min at 37 °C. The final concentration of the enzyme in the assay was 0.25 μM, and the final concentration of the inhibitors was 0.4 to 53 μM (Mcp2a) or 0.8 to 105 μM (Mcp4a). The velocity of substrate turnover was calculated as fluorescence units/s, and the data points were fitted to the Morrison equation using the GraphPad Prism program (version 5.0). All experiments were carried out at least in triplicate.

### Testing cleavage and religation of mycocypins

To test if legumain processes mycocypins, it was incubated with mycocypins in a 1:2 molar ratio for 1 h at 37 °C in a buffer composed of 50 mM NaCl and 20 mM citric acid pH 4.0, 5.0, or 6.0 or 50 mM NaCl and 20 mM Tris pH 7.0. For the Clt2-T71C mutant, a molar ratio of 1:3 was used and incubation was done on ice. Progress of inhibitor hydrolysis was monitored on SDS-PAGE with or without preheating of the samples at 95 °C for 20 min.

To test whether mycocypins were also a substrate to legumain’s ligase activity, we incubated Mcp1a or Clt2 with legumain in a 1:2 molar ratio at pH 4, for 1 h at 37 °C. Subsequently we shifted pH to neutral by adding 100 mM of Tris pH 7.0 or we added 2 mM of MMTS to the sample and further incubated the reactions for 1 h at 37 °C. Progress of cleavage and religation was monitored on SDS-PAGE.

### Thermal stability assay

The thermal stability of legumain upon incubation with different mycocypins was assayed using differential scanning fluorimetry. Briefly, legumain was mixed with different mycocypin variants in a 1:1 molar ratio at 0.1 mg/ml final protein concentration, in an assay buffer composed of 100 mM citric acid pH 6.0, 100 mM NaCl, and 5x Sypro orange Dye (Invitrogen). Thermal unfolding was measured as an increase in fluorescence signal in an 7500 Real Time PCR System (Applied Biosystems) after increasing temperature by 1 °C per min from 20 °C to 95 °C. Fluorescence data was normalized to peak values and melting curves were evaluated as described previously (9).

### Analysis of complex formation

Complex formation of legumain with mycocypins was further analyzed by comigration assays. Specifically, legumain was mixed with Mcp1a, Mcp3a, or Clt2 in a 1:2 M ratio in assay buffer containing 20 mM citric acid pH 4.0 and 50 mM NaCl for 30 min on ice. Subsequently, the mix was loaded on a Superdex S75 10/300 Gl column preequilibrated in assay buffer, to separate complexed from free inhibitors. Control experiments contained only the enzyme or only the inhibitor. Peak fractions were analyzed by SDS-PAGE.

Cleaved Clt2 inhibitor was prepared by incubating legumain and Clt2 in a 1:4 molar ratio in a buffer composed of 20 mM citric acid pH 5.5 and 50 mM NaCl for 4 h at 37 °C. To separate legumain from excess, cleaved inhibitor, the mixture was loaded on a Superdex S75 10/300 Gl column preequilibrated in a buffer containing 20 mM Tris pH 7.5 and 50 mM NaCl.

### SDS-PAGE analysis

Proteins were separated on 16.5% SDS-PAGE gels after addition of an appropriate amount of reducing or nonreducing 4x-SDS loading buffer (40% glycerol, 8% SDS, 200 mM Tris pH 6.8, and 0.16% bromophenol blue). Gels were stained using Coomassie Brilliant Blue-G (panreac applichem). Molecular weights were assigned using the PageRuler prestained protein ladder as a reference (10–250 kDa, Thermo Scientific). Mycocypins proved to be resistant to unfolding in SDS-loading buffer. Therefore, increased concentrations of SDS (up to 15%) or urea (3 M) were added, if specified.

### Mass spectrophotometry

Legumain was incubated with Mcp1a or Clt2 in a 1:1 molar ratio in a buffer composed of 20 mM citric acid pH 4 and 50 mM NaCl for 30 min at 37 °C. Subsequently, samples were analyzed by SDS-PAGE and mass spectrometry, utilizing an ESI-Orbitrap setup. Similarly, Kgp was incubated with Mcp1a-N74K or Mcp1a-I72A-N74K in a 1:10 molar ratio in Kgp assay buffer at 37 °C for 3 h and analyzed by SDS-PAGE and mass spectrometry. For mass spectrometric analysis, samples were desalted with C18 ZipTips (Merck Millipore), eluted from the tips with 50% acetonitrile in 0.1% formic acid and directly infused into the mass spectrometer (Q Exactive Orbitrap mass spectrometer, Thermo Fisher Scientific) at a flow rate of 1 μl/min. Capillary voltage at the nanospray head was 2 kV. Raw data were processed with Protein Deconvolution 2.0 (Thermo Fisher Scientific). Masses were assigned to the protein sequence with the Protein/Peptide Editor module of BioLynx (part of MassLynx V4.1, Waters).

### Sequence alignment

The sequences of different mycocypins were obtained from the UniProtKB/Swiss-Prot databases. The sequences we used include *C. nebularis* clitocypin 2 (Q9P4A2), *M. procera* macrocypin 1a (B9V973), macrocypin 1b (B9V975), macrocypin 1c (B9V976), Mcp2a (B9V977), macrocypin 2b (B9V978), macrocypin 3a (B9V979), macrocypin 3b (B9V980), macrocypin 3c (B9V981), macrocypin 4a (B9V982), macrocypin 4b (B9V983), and macrocypin 5a (B9V984). A sequence alignment was prepared using Clustal W2 (43) and further modified using structure-based alignments prepared with TopMatch (44). For visualization of the alignments, we used the ALINE sequence editor program (45).

### Crystallization and X-ray data collection of protein complexes

For complex formation, legumain and Mcp1a were mixed in a 1:1.1 molar ratio in a buffer composed of 20 mM citric acid pH 5.5, 50 mM NaCl, and 0.5 mM MMTS. The reaction was incubated for 30 min on ice and subsequently concentrated to a final concentration of approximately 20 mg/ml of the complex utilizing Vivaspin concentrators (MWCO: 10 kDa, Sartorius Stedim Biotech). Initial crystallization screening was performed in a sitting-drop vapor diffusion setup. 0.2 μl concentrated enzyme–inhibitor complex were mixed with 0.2 μl JBScreen Classic (Jena Bioscience) screen solution and equilibrated against 60 μl reservoir solution in 96 well INTELLI-PLATEs at 4 °C. After approximately 1 week, crystals appeared in a condition composed of 1.6 M ammonium sulfate and 500 mM LiCl, pH 5.2. For cryo-protection, a cryo-solution composed of the reservoir solution supplemented with 30% glycerol was added stepwise to the drops containing crystals before flash freezing in liquid nitrogen. A native x-ray diffraction data set was collected at beamline ID30A-3 (ESRF Grenoble) at a wavelength of 0.9677 Å and 0.1 ° oscillation range to a resolution of 2.2 Å.

Similarly, the legumain–Clt2 complex was formed by incubation of legumain with Clt2 in a 1:1.1 molar ratio in a buffer composed of 20 mM citric acid pH 5.5, 50 mM NaCl, and 0.5 mM MMTS. After concentrating the complex to approximately 20 mg/ml final protein concentration, initial crystallization screening experiments were set up using the Hampton index screen (Hampton). After approximately 2 weeks, at 4 °C, we obtained an initial hit in a condition composed of 0.1 M sodium citrate pH 4.5 and 20% PEG 4000. Crystals were optimized by fine-screening around the originally identified condition and flash frozen in liquid nitrogen after incubation in a cryo-solution composed of the original condition supplemented with 30% ethylene glycol. An x-ray diffraction data set was collected at the ESRF in Grenoble at beamline ID30A-3 at a wavelength of 0.9677 Å and 0.2° oscillation range to a resolution of 1.8 Å.

### Structure solution

X-ray diffraction data was processed utilizing XDS (46). An initial model was obtained by molecular replacement using AutoMR from the Phenix (Python-based Hierarchical Environment for Integrated Xtallography) program suite utilizing coordinates of legumain and Mcp1a or Clt2. Iterative cycles of rebuilding in COOT (47) followed by refinement in phenix.refine (48) and REFMAC (49) were carried out. The final structures were analyzed using PROCHECK (50) and MolProbity (51). Coordinates and structure factors were deposited with the PDB under entry codes 8AE5 and 8AE4. Pymol (52) was used to create figures illustrating structures. Complex assemblies of legumain with Mcp1a or Clt2 were analyzed using the ‘Protein interfaces, surfaces, and assemblies’ service PISA (53) at the European Bioinformatics Institute (https://www.ebi.ac.uk/pdbe/pisa/).

### Molecular modelling

Models of mycocypins in complex with *P. gingivalis* Kgp, human cathepsin V, and *A. thaliana* legumain isoforms β and γ were prepared by superposing structures of human legumain in complex with Mcp1a or Clt2 onto structures of Kgp (pdb 4rbm), the Clt2-cathepsin V complex (pdb 3h6s), AtLEGβ (pdb 6ysa), and AtLEGγ (5obt) using TopMatch (44). Pymol was used for visualization (52).

## Data availability

The structures presented in this article have been deposited in the Protein Data Bank (PDB) with the accession codes 8AE4 (legumain in complex with clitocypin 2) and 8AE5 (legumain in complex with macrocypin 1a).

## Supporting information

This article contains supporting information (43, 44, 45).

## Conflict of interest

The authors declare that they have no conflict of interest with the contents of this article.
