# Supplementary material for: Structural and functional studies of legumain–mycocypin complexes revealed a competitive, exosite-regulated mode of interaction
Source: J Biol Chem. 2022 Sep 16;298(10):102502. doi: 10.1016/j.jbc.2022.102502 (PMC9579014; doi:10.1016/j.jbc.2022.102502)
Supplement: Supporting information [file mmc1.docx]

**Supporting Information**

**Structural and functional studies of legumain-mycocypin complexes revealed a competitive, exosite-regulated mode of interaction**

Tasneem Elamin^#^, Naiá P. Santos^#^, Peter Briza^#^, Hans Brandstetter^#^, Elfriede Dall^#†^

**Affiliations:**

^#^Department of Biosciences and Medical Biology, University of Salzburg, 5020 Salzburg, Austria.

**Corresponding Author:**

^†^Elfriede Dall (elfriede.dall@plus.ac.at)

**Supplemental Discussion**

The crystal structure of the AEP-Mcp1a complex revealed two assemblies of the complex within the asymmetric unit (Fig. S1A). Both complexes were interacting within the asymmetric unit, primarily via interactions of the two Mcp1a molecules. These interactions included e.g. hydrophobic contacts of Tyr141 to Met27 and hydrogen bonding interactions of Glu143 and Asp13 to the amide nitrogens of Gly142 and Tyr141. Within the crystal, the Mcp1a molecules mediated tight packing. Four Mcp1a molecules from 2 asymmetric units assembled in another larger subunit that was building up the crystal. This tetrameric assembly of the AEP-Mcp1a complex was stabilized by tight interactions of the Mcp1a molecules. These interactions were mostly hydrophobic and included Ile129 – Val127, Val153 – Ile129 and Ile154 – Ile154.

The crystal structure of the AEP-Clt2 complex contained one complex assembly within the asymmetric unit (Fig. S1B). Crystal contacts were established by Clt2, which was mediating contacts to neighboring AEP molecules from adjacent asymmetric units. Furthermore, the glycan chain linked to Asn91 on legumain was interacting with residues Phe135, Lys133 and Asn85 on a neighboring Clt2 molecule. Further crystal contacts were established by Arg116 on Clt2 which was mediating hydrophobic interactions to Tyr220, Trp232 and Cys219 on legumain and by a salt bridge formed by Asp20 on Clt2 and Arg34 on a neighboring Clt2 molecule. To crystallize legumain in complex with Clt2 we used a legumain-N263Q mutant, which was lacking glycosylation on residue Asn263. Gln263 was involved in a crystal contact to Gln261 of another legumain molecule. The legumain-N263Q glycosylation mutant was important for crystallization, as the stabilizing interaction established by the mutant would have been abolished by the glycosylation if wild-type legumain was used in the crystallization experiment.

**Supplementary Figure 1. Crystal packing of the AEP-Mcp1a (A) and AEP-Clt2 (B) complexes.** Legumain is shown in green, Mcp1a in purple and Clt2 in orange. Asymmetric units are indicated by a white rectangle.

**Supplementary Figure 2. Mycocypins show a substrate-like mode of binding to legumain.** A) Zoom-in view on the active site of legumain (green) bound to Mcp1a (purple). B) Zoom-in view on the active site of legumain (green) bound to Clt2 (orange). A 2Fo-Fc composite omit electron density map contoured at 2 σ over the mean is shown surrounding the RCL sequences.

**Supplementary Figure 3. Structure-based sequence alignment of mycocypins.** Mycocypin sequences were obtained from the UniProtKB/Swiss-Prot database. The sequences used are *Clitocybe nebularis* clitocypin 2 (Q9P4A2), *Macrolepiota procera* macrocypin 1a (B9V973) macrocypin 1b (B9V975), macrocypin 1c (B9V976), macrocypin 2a (B9V977), macrocypin 2b (B9V978), macrocypin 3a (B9V979), macrocypin 3b (B9V980), macrocypin 3c (B9V981), macrocypin 4a (B9V982), macrocypin 4b (B9V983) and macrocypin 5a (B9V984). The sequence alignment was prepared using Clustal W2 (54) and further modified using structure superimpositions prepared with TopMatch (55). Secondary structure elements are assigned in purple for Mcp1a (pdb 3h6q) and in orange for Clt2 (pdb3h6r). Residues forming exosite 1 and exosite 2 in Mcp1a are labelled with purple diamonds, the P1-Asn residue on the reactive center loop (RCL) is highlighted by a red star and the papain inhibitory G25G26-motif is labeled with black rectangles. The sequence editor program Aline was used to visualize the alignment (56).

**Supplementary Figure 4. Legumain cleaves mycocypins after the P1-Asn residue on the reactive center loop.** A) legumain was co-incubated with Clt2 in a 1:2 molar ratio at indicated pH values for 1 h at 37 °C. Subsequently samples were taken, mixed with non-reducing SDS-loading buffer and heated to 95 °C for 20 minutes. B) Same as A, but legumain was mixed with Mcp1a. C) and D) Indicated mycocypin variants were loaded on SDS-PAGE, in reducing or non-reducing loading buffer, with or without heating of the samples at 95 °C. E) Intact masses determined for Clt2 alone and Clt2 after incubation with legumain at pH 4.0. Peaks corresponding to intact Clt2 (2-160) and P1-Asn71^Clt^ processed Clt2 could be identified. N-terminal cleavage product: 2-70, C-terminal cleavage product: 71-160. F) Intact mass of legumain co-incubated with Mcp1a at pH 4.0 in a 1:2 molar ratio. The spectrum revealed intact Mcp1a (1-177 and 2-177) and the N-terminal (1-74 and 2-74) and C-terminal (75-177) cleavage products resulting from cleavage after P1-Asn74^Mcp^ on the RCL.

**Supplementary Figure 5. Cleaved mycocypins remain bound to legumain and are not substrates to its ligase activity.** A) Legumain (AEP) alone, Clt2 alone or legumain co-incubated with Clt2 (AEP-Clt2) were loaded on an S75 column pre-equilibrated in buffer at pH 4.0. Peak fractions of legumain co-incubated with Clt2 were loaded on the SDS-PAGE gel. The first peak contained legumain and processed Clt2 inhibitor, confirming stable complex formation. B) Similar as (A), but Mcp1a was used. The first peak contained legumain, intact and processed Mcp1a. C) To test whether processed Mcp1a* and Clt2’ were substrates to legumain’s ligase activity, we co-incubated legumain with Mcp1a or Clt2 in a 1:2 molar ratio at pH 4.0 for 1 hour at 37 °C, to generate processed Mcp1a* and Clt2*. Subsequently the reactions were shifted to pH 7.0 or MMTS was added to trigger ligase activity. No conversion of Mcp1a* or Clt2* to the intact forms was observed.

**Supplementary Figure 6. The conformation of the Mcp1a RCL is critical for inhibition.** A) Legumain was co-incubated with Mcp1a-D73A and Mcp1a-R71E variants at indicated pH values for 1 hour at 37 °C. Subsequently samples were analysed by SDS-PAGE (without heating). Cleavage of the Mcp variants was observed at pH 4.0 to 6.0. B – D) To determine K_i_ values of Mcp1a wild-type and the Mcp1a-D73A and Mcp1a-R71E mutants towards legumain, initial velocities were fitted to the Morrison equation using the GraphPad Prism program (version 5.0, La Jolla, CA). All experiments were carried out at least in triplicate. E) The thermal stability of legumain was tested at pH 6.0 after adding Mcp1a, Mcp1a-D73A and Mcp1a-R71E variants. Melting points are indicated by dashed lines.

**Supporting Figure 7. Mycocypins harboring a cysteine in P1’ position can serve as covalent inhibitors of legumain.** A) Legumain was incubated with Mcp3a at indicated pH values at 37 °C for 1 hour. Subsequently samples were taken, mixed with non-reducing loading buffer and subjected to heating to 95 °C for 20 min if indicated, before analyzing them on SDS-PAGE. B) Same as in (A), but samples were mixed with reducing SDS-loading buffer. In the absence of a reducing agent, Mcp3a forms a disulfide mediated, covalent dimer, in the loading buffer. C) Size exclusion chromatography experiments of legumain alone (AEP), Mcp3a alone and legumain pre-incubated with Mcp3a (AEP-Mcp3a). Samples were analyzed on an S75 column equilibrated in buffer at pH 5.5. Peak fractions were analyzed by SDS-PAGE using non-reducing or reducing sample loading buffer. The covalent complex migrated as a separate peak which was reduced to AEP and Mcp3a bands in the presence of a reducing agent. D) Enzymatic activity of legumain incubated with Mcp1a or Mcp1a-S75C measured at pH 4.0 or pH 5.5. E) Co-incubation of legumain (AEP) with Clt2 wild-type and Clt2-T71C inhibitors at pH 4.0 – 7.0. Samples were heated to 95 °C before loading on SDS-PAGE gels. Covalent complex formation was observed at pH 5.0, 6.0 and 7.0 and the complex was resolved under reducing (+DTT) conditions (F). Additionally, we observed a disulfide mediated Clt2-T71C dimer, which was also resolved in the presence of DTT. G) Enzymatic activity of legumain (2 nM) towards the AAN-AMC substrate was measured at pH 5.5 after addition of 2 µM Clt2 variants in assay buffer with or without 2 mM DTT. H) Enzymatic activity of legumain after incubation with 10 nM Mcp1a wild-type or the Mcp1a-S75A mutant measured at pH 5.5.

**Supplementary Figure 8. The exosite interaction stabilize the legumain-inhibitor complexes.** A) Stereo view on the active site of human legumain (green) bound the Mcp1a (purple). Regions forming the RCL and exosite 1 and 2 are indicated. B) Cleavage assay showing that the Mcp1a-R96A mutant is processed efficiently by legumain at pH 4.0 to 6.0 (Mcp1a-R96A*: processed Mcp1a-R96A). C) Thermal unfolding of legumain, measured at pH 6.0 in the presence or absence of Mcp1a or the Mcp1a-R96A mutant. Melting temperatures are indicated by dashed lines. D) Cleavage assay showing that the Clt2-3x mutant is processed efficiently by legumain at pH 4.0 to 6.0 (Clt2-3x*: processed Clt2-3x). E) Thermal unfolding of legumain, measured at pH 6.0 in the presence or absence of Clt2 or Clt2-3x mutant. Melting temperatures are indicated by dashed lines. F) Stereo view on the active site of human legumain (green) bound to Clt2 (orange). Regions forming the RCL and exosite 1 and 2 are indicated.

**Supplementary Figure 9. Models of mycocypins in complex with *Arabidopsis thaliana* legumain isoforms β (AtLEGβ) or γ (AtLEGγ).** A) Model of AtLEGβ (dark blue) and AtLEGγ (light blue) in complex with Mcp1a (purple). Models were prepared by superposing the structure of human legumain (grey) in complex with Mcp1a on the structures of AtLEGβ (pdb 6ysa) or AtLEGγ (pdb 5obt). Potentially interacting residues are shown as sticks. B) Model of AtLEGβ (dark blue) and AtLEGγ (light blue) in complex with Clt2 (orange). The model was prepared similar as in (A) but using the structure of human legumain in complex with Clt2 for superposition.

**Supplementary Figure 10. *Porphyromonas gingivalis* gingipain K (Kgp) is inhibited by Mcp2a variants.** A) Model of *Porphyromonas gingivalis* gingipain K (Kgp) bound to the Mcp2a reactive center loop (RCL). The model was prepared by superposing the legumain-Mcp1a complex structure onto the Kgp structure (pdb 4rbm) and *in silico* mutation of the RCL sequence to the Mcp2a sequence. Kgp is shown in blue, the Mcp2a RCL as purple sticks. B) Kgp is inhibited by Mcp2a but even more potently by the Mcp2a-S75C variant, suggesting covalent complex formation to the catalytic cysteine residue. The enzymatic activity of 0.25 µM Kgp after incubation with 1.5 µM of the respective Mcp2a variant was measured as turnover of the chromogenic Tos-Gly-Pro-Lys-pNA (Bachem) substrate.

**Supplementary Figure 11. The scissile peptide bond is slightly shifted in Mcp1a and Clt2 relative to cystatin E (hCE).** Superposition of the crystal structures of legumain (green) in complex with human cystatin E (4n6o), Clt2 or Mcp1a. The reactive center loops of the inhibitors are shown in grey (hCE), purple (Mcp1a) or orange (Clt2). Catalytic residues are shown in blue sticks, residues forming the S1-pocket in green sticks.
